# A Sequence in Subdomain 2 of DBL1α of *Plasmodium falciparum* Erythrocyte Membrane Protein 1 Induces Strain Transcending Antibodies

**DOI:** 10.1371/journal.pone.0052679

**Published:** 2013-01-15

**Authors:** Karin Blomqvist, Letusa Albrecht, Maria del Pilar Quintana, Davide Angeletti, Nicolas Joannin, Arnaud Chêne, Kirsten Moll, Mats Wahlgren

**Affiliations:** 1 Department of Microbiology, Tumor and Cell Biology (MTC), Karolinska Institutet, Stockholm, Sweden; 2 Escuela de Medicina y Ciencias de la Salud, Facultad de Ciencias Naturales y Matemáticas, Universidad del Rosario, Bogotá, Colombia; 3 Bioinformatics Center, Institute for Chemical Research, Kyoto University, Kyoto, Japan; 4 Biologie des interactions Hôte-Parasite, Institut Pasteur, Paris, France; University of Oklahoma Health Sciences Center, United States of America

## Abstract

Immunity to severe malaria is the first level of immunity acquired to *Plasmodium falciparum*. Antibodies to the variant antigen PfEMP1 (*P. falciparum* erythrocyte membrane protein 1) present at the surface of the parasitized red blood cell (pRBC) confer protection by blocking microvascular sequestration. Here we have generated antibodies to peptide sequences of subdomain 2 of PfEMP1-DBL1α previously identified to be associated with severe or mild malaria. A set of sera generated to the amino acid sequence KLQTLTLHQVREYWW**ALNRKE**VWKA, containing the motif ALNRKE, stained the live pRBC. 50% of parasites tested (7/14) were positive both in flow cytometry and immunofluorescence assays with live pRBCs including both laboratory strains and *in vitro* adapted clinical isolates. Antibodies that reacted selectively with the sequence REYWWALNRKEVWKA in a 15-mer peptide array of DBL1α-domains were also found to react with the pRBC surface. By utilizing a peptide array to map the binding properties of the elicited anti-DBL1α antibodies, the amino acids WxxNRx were found essential for antibody binding. Complementary experiments using 135 degenerate RDSM peptide sequences obtained from 93 Ugandan patient-isolates showed that antibody binding occurred when the amino acids WxLNRKE/D were present in the peptide. The data suggests that the ALNRKE sequence motif, associated with severe malaria, induces strain-transcending antibodies that react with the pRBC surface.

## Introduction

The protozoan parasite *Plasmodium falciparum* infects some 225 million people annually and causes the death of ≈ 1 million individuals, mainly children under the age of five and pregnant women [1]. In order to establish persistent blood stage infections, *P. falciparum* undergoes antigenic variation. The major variant antigen PfEMP1 (*P. falciparum* erythrocyte membrane protein 1) is expressed at the red blood cell (RBC) surface and is encoded by the *var* gene family with around 60 copies per haploid genome [2], [3], [4]. PfEMP1 is a virulence associated adhesion molecule that participates in the causation of severe malaria by mediating the accumulation of parasitized- and unparasitized erythrocytes in the microvasculature through endothelial binding (cytoadhesion) and through binding to unparasitized erythrocytes (rosetting) [5]. The NTS-DBL1α domain of PfEMP1 has been identified both as a ligand for rosetting and cytoadhesion involving receptors such as heparan sulfate, complement receptor 1 and blood group A [6], [7], [8].

Acquired immunity to malaria develops in individuals living in endemic areas, after recurrent exposure to the parasite, but it is not sterilising [9], [10], [11]. Likewise, women become resistant to pregnancy-associated malaria after repeated pregnancies and infections but the presence of scanty low-grade parasitaemia is also seen in the immune [12], [13]. Immunity to severe disease is the first level of protection that develops in endemic areas and it is associated with the presence of antibodies to variable surface antigens such as PfEMP1 at the parasitized red blood cell (pRBC) surface [14], [15], [16], [17]. Such antibodies confer protection by preventing the sequestration of pRBC (rosetting and cytoadherence) and by opsonizing pRBCs for phagocytosis [12], [18], [19], [20], [21].

It is not understood how immunity to the highly variable PfEMP1 antigen develops. There are two main hypotheses: one suggests that immunity consists of strain-transcending, cross-reactive antibodies to a few highly conserved epitopes whereas the other implies that immunity is comprised of a large pool of more strain-specific antibodies. Early indications that immunity is strain-specific came from studies on malaria infection as a treatment for neurosyphilis [22], and there are also studies using *in vitro* adapted laboratory *P. falciparum* strains, that indicate antibodies to PfEMP1 to react in a strain-specific manner [18], [20]. However, clinical data suggest that patients rapidly acquire immunity that protects against severe disease [14], [15], [16], [17] and varying degrees of cross-reactivity have been demonstrated on a serological level using either sera from malaria infected individuals on heterologous parasites or sera from PfEMP1-immunized animals on heterologous PfEMP1 proteins or parasites [23], [24], [25], [26], [27], [28], [29].

By studying the *var* gene transcription profiles of fresh clinical *P.* *falciparum* isolates of 93 Ugandan children we have previously demonstrated a correlation between different degenerate PfEMP1-DBL1α amino acid motifs and different states of malaria [30]. The collected sequences were examined with a novel method of region alignment of homology areas (MOTIFF) to identify degenerate sequence motifs correlated with specific disease states. The key DBL1α motifs identified were found also expressed in *in vitro* adapted strains. We hypothesized that subgroups of cross-reactive PfEMP1-DBL1α sequences are exposed on the pRBC surface and are recognized by the immune system leading to the development of cross-reactive antibodies that protect against severe malaria. In this paper we show that the earlier identified sequence motifs can induce antibodies and that some of them react with the surface of the live pRBC of different parasite isolates as well as *in vitro* adapted strains in a strain-transcendent fashion.

## Results

### Generation of Specific Antibodies Towards NTS-DBL1α Sequence Motifs

A set of peptides covering six of the degenerate PfEMP1 sequence motifs that were associated with severe or mild malaria in clinical isolates from Uganda [30] were produced (Table 1, Fig. S1 and S2). Of the six peptides, four were from motifs associated with severe malaria and two were from motifs associated with mild malaria. The peptide sequences and the corresponding parasite isolate expressing the particular motif are listed in Table 1. Two rabbits and one rat were immunized per peptide. All of the six DBL1α -motif peptides induced antibodies in both species as confirmed in ELISA with the corresponding peptide (not shown). After an initial screen on live pRBCs using flow cytometry (FACS) and immunofluorescence assays (IFA) on air-dried monolayers of pRBCs, a subset of six sera (rabbit 1 and 2 for DBL1α-S1S2; rabbit 2 and rat for DBL1α-RDSM; rabbit 1 and rat for DBL1α-L1M4) showing positive results were affinity purified on the corresponding peptide using the Ultralink Iodoacetyl matrix (see Material and Methods).

**Table 1:** Synthesized peptides.

Motif^a^	Motif name	Peptide sequence^b^	Expressed in parasite
**EPGIQHLEK**	**S1S2**	HLRHEPGIQHLEKRLESMFQNIQN	**UAS22**
**ALNRKE**	**RDSM** c **(S3)**	KLQTLTLHQVREYWWALNRKEVWKA	**UAS22**
**TCGATM**/**FSKNI**	**H1H2**	ITCGATMNDIFSKNIR	**UAS22**
**NDEVWK**	**H3**	TDNDEVWKGLRAVFGKI	**UAS31**
**ICTVL**	**M5**	SNICTVLARSFADIGDIV	**UAS29**
**DSIKKY**	**L1/M4**	EGDSIKKYHDKHQGT	**UAS29**

a Identified by Normark et al [30].

b The motifs are underlined in the peptide sequences.

c The motif is named RDSM (Respiratory distress severe malaria) in this article to avoid confusion with the S3 domain of DBL1α [35].

### Immunofluorescence Assay on Air-dried Monolayers of pRBC

**Figure 1:**
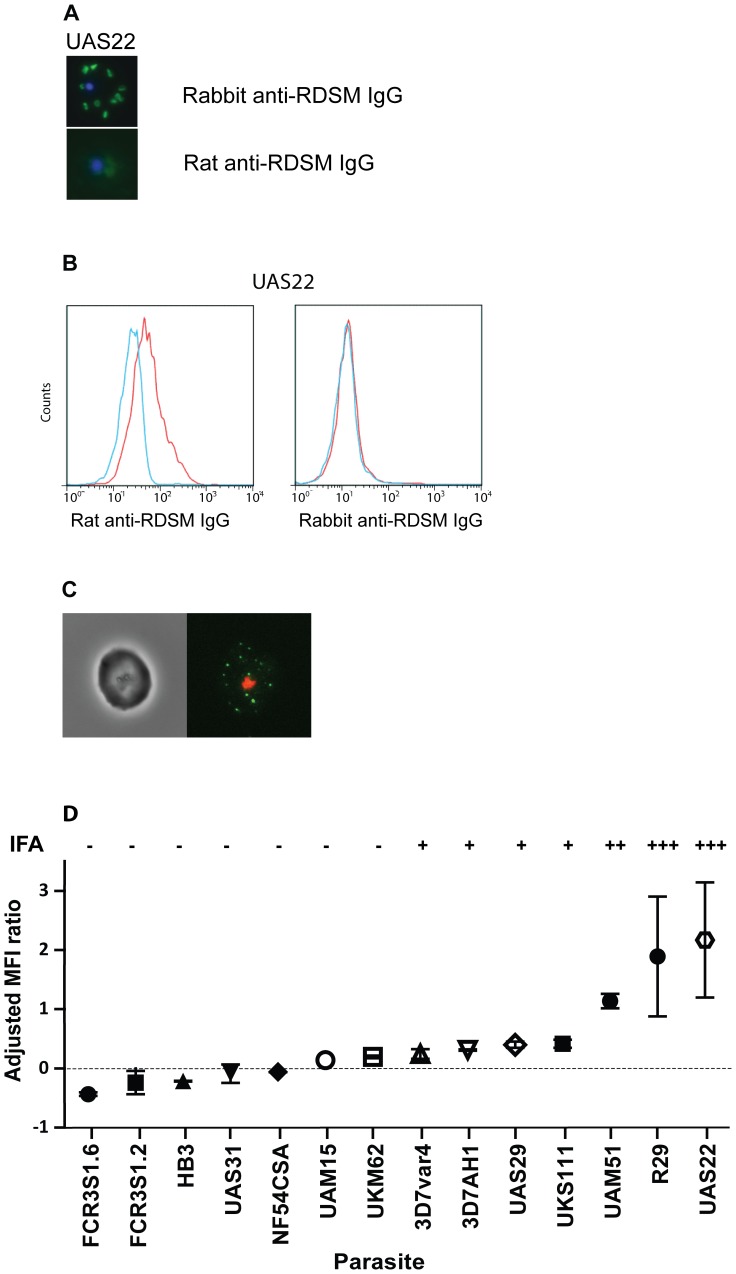
The DBL1α-RDSM peptide induces strain-transcending antibodies that react with the pRBC surface. A. Indirect IFA on air-dried monolayers of the clinical isolate UAS22. Specific donut pattern shown with rabbit anti-DBL1α-RDSM IgG (green) while rat anti-DBL1α-RDSM IgG only stained the internal parasite. The parasite nucleus is stained with DAPI (blue). The rat anti-DBL1α-RDSM IgG is from rat 1. B. Analysis of surface recognition by FACS. Rat anti-DBL1α-RDSM IgG (red) assayed on the parasites UAS22 and FCR3S1.2. Non-immune rat IgG at the same concentration is shown in blue. The rat anti-DBL1α-RDSM IgG is from rat 1. C. Live cell immunofluorescence microscopy. Rat anti-DBL1α-RDSM IgG binding to UAS22. The parasite nucleus is stained with ethidium bromide (red). The rat anti-DBL1α-RDSM IgG is from rat 1. D. Analysis of surface recognition by FACS and live cell immunofluorescence in 14 isolates and strains with rat anti-DBL1α-RDSM IgG. The adjusted MFI ratio ± SEM is shown. The corresponding results for immunofluorescence assays (IFA) on live parasites are also shown.

All affinity purified peptide antibodies were assayed on air-dried monolayers for binding to pRBCs from a panel of Ugandan parasites and laboratory strains. IgG from the rabbit immunized with the DBL1α-RDSM (respiratory distress severe malaria) peptide showed a donut shape fluorescence pattern (Fig. 1A), typical for exported proteins like PfEMP1 associated with Maurer’s clefts in the red blood cell cytosol [31]. The rabbit anti-DBL1α-RDSM IgG showed the same pattern for all parasite strains and isolates screened (UAS22, FCR3S1.2, R29, UAS29, 3D7AH1 and UKS111, Fig. S3). The rat immunized with the DBL1α-RDSM peptide (Fig. 1A) as well as the other affinity purified antibodies showed reactivity with the internal parasite only.

### Recognition of pRBC Surface Exposed Epitopes by Anti-DBL1α Motif Antibodies

**Figure 2:**
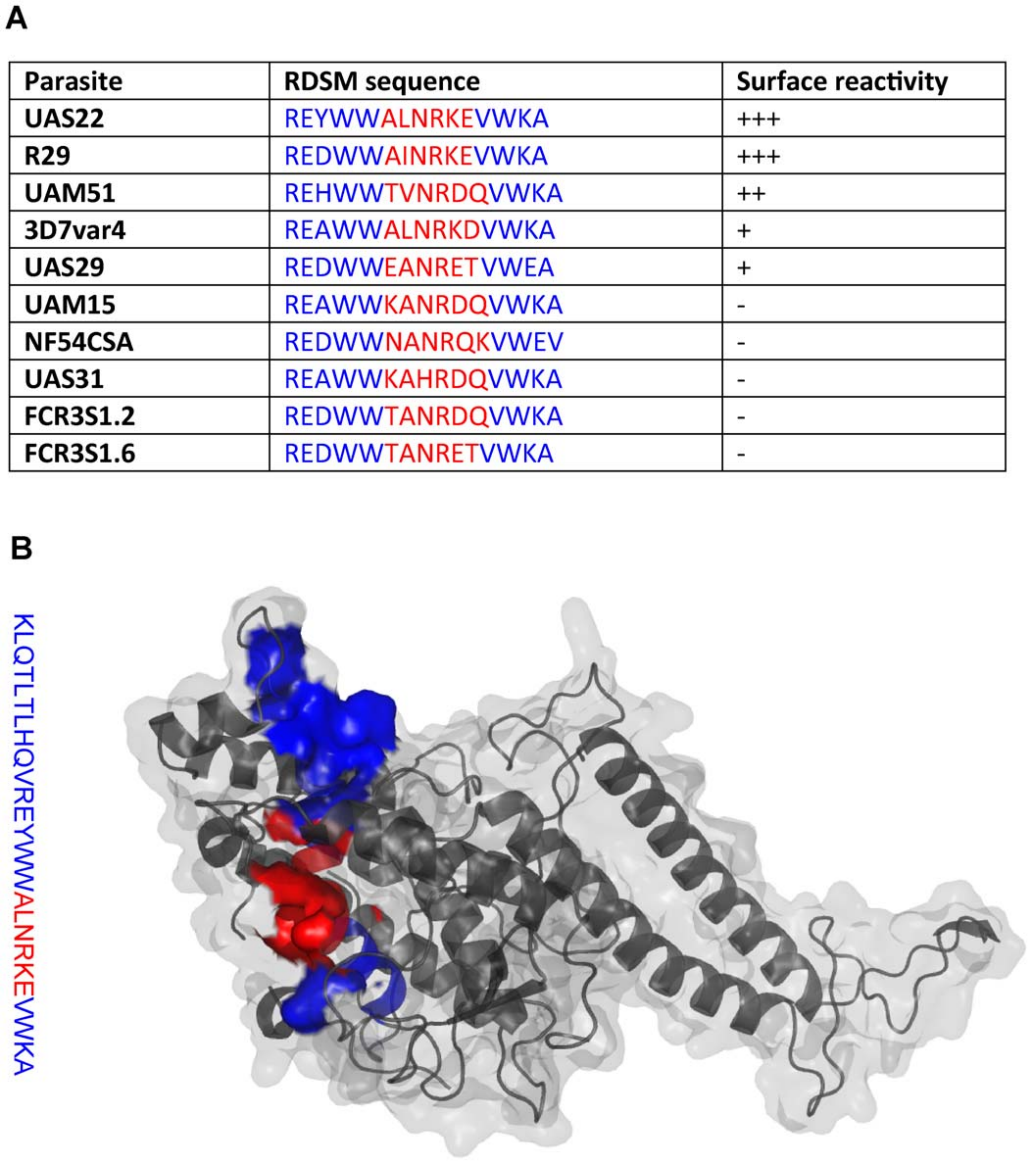
RDSM amino acid sequence and location within the PfEMP1-DBL1α domain. A. Surface recognition correlated with DBL1α-RDSM amino acid sequence. Only strains and isolates with known or predicted (by RT-PCR) DBL1α amino acid sequence are included. B. Location of the RDSM peptide within the predicted crystal structure of the R29 PfEMP1-DBL1α domain. The blue part depicts the RDSM peptide and the red part the RDSM motif. The R29 sequence is modeled on the crystallized Palo Alto_varO_ NTS-DBL1α.

The surface reactivity of the set of six anti-peptide antibodies was analyzed using FACS. Most antibody-preparations were negative in FACS, including rabbit anti-DBL1α-RDSM antibodies, but rat anti-DBL1α-RDSM antibodies stained the surface of pRBCs parasitized with the UAS22 parasite strain (the homologous parasite from which the RDSM peptide sequence was obtained; Fig. 1B). In live cell fluorescence microscopy, these antibodies showed a dotty fluorescence pattern (Fig. 1C), typical for PfEMP1-staining of the parasitized erythrocyte surface [20], [28], [32]. To further explore the reactivity of the anti-DBL1α motif antibodies, a panel of seven recently established isolates from Ugandan children and seven long term propagated strains (Tables 2 and 3) were screened in FACS and in live cell IFA. The rat anti-DBL1α-RDSM antibodies showed surface reactivity both in FACS and in live cell IFA with seven out of 14 parasites studied, (Fig. 1D). Four parasites, UKS111, UAS29, 3D7AH1 and 3D7var4 reacted moderately while three parasites, UAS22, R29 and UAM51, showed stronger surface reactivity than the others. The latter three parasites are all shown to express a PfEMP1 with a DBL1α domain containing an amino acid motif similar to that used for immunization. Figure 2A shows the correlation between the surface reactivity in FACS and the demonstrated amino acids in the RDSM sequence of the screened parasite strains and isolates. Figure 2B depicts the RDSM peptide location within a NTS-DBL1α model of the parasite strain R29 based on the crystallized NTS-DBL1α-domain of the Palo Alto_varO_ parasite line.

**Table 2:** Clinical isolates included in the study.

Clinical isolate	Sex	Age (months)	Clinical diagnosis	Initial rosetting rate (%)	*In vitro* rosetting rate (%)	Parasite generations at FACS/IFA^d^
UAS22	M	17	Respiratory distress	47	<5	20–24
UAS29	F	12	Respiratory distress	9	10	14–18
UAS31	M	30	Severe malaria NUD	56	20	10–14
UKS111	F	30	Cerebral malaria	50	40	1–4
UKM62	M	15	Mild malaria	<5	<5	9–10
UAM15	M	24	Mild malaria	2	<5	15–18
UAM51	F	6	Mild malaria	4	<5	15–18

d Dominant *var* gene determined by reverse transcriptase-PCR at zero generations for UAS22, UAS29, UAS31, UAM15 and UAM 51 as published in Normark *et al* [30].

**Table 3:** Laboratory strains included in the study.

Laboratory strain	Rosetting rate (%)	Selection/enrichment
FCR3S1.2	>80	mAb enrichment
FCR3S1.6	<5	–
R29	70	mAb enrichment
HB3	0	–
3D7AH1	<5	–
3D7var4	<5	mAb enrichment
NF54CSA	<5	CSA panning

### Immunogenicity of the RDSM Peptide

**Figure 3:**
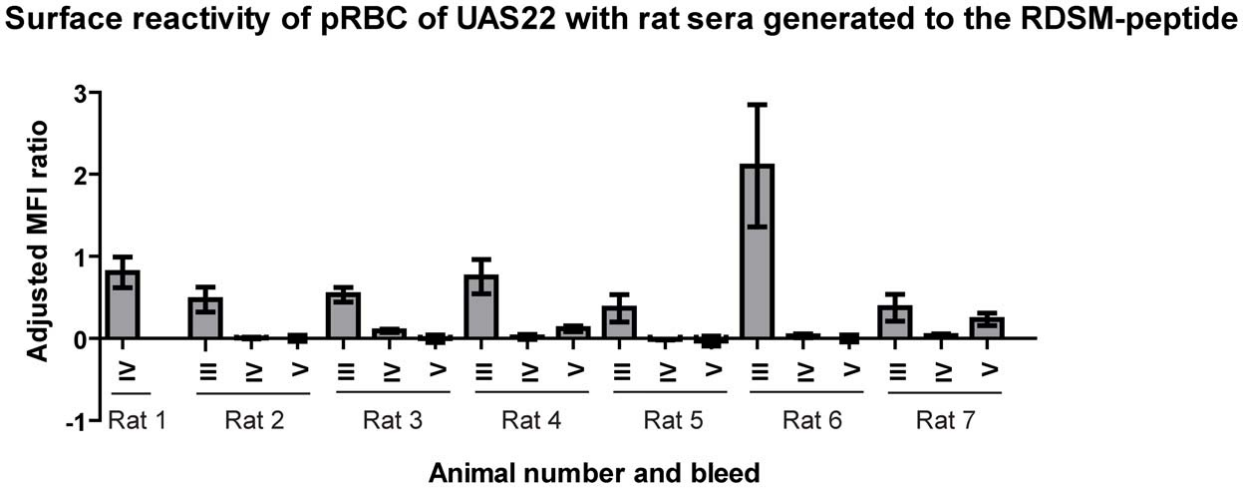
Surface reactivity of anti-DBL1α-RDSM rat sera to pRBCs of UAS22. FACS results from sera after third immunization assayed on UAS22 in trophozoite stage. For comparison, serum from rat 1 (fourth immunization) is included as a reference. The adjusted MFI ratio ± SEM is shown.

We further explored the immunogenicity of the RDSM peptide by immunizing another set of six rats. Five immunizations were carried out and all animals showed reactivity in FACS after the third immunization (Fig. 3). This relatively strong reactivity decreased with subsequent immunizations but after the last immunization, five out of six rat sera (named rat sera 2–7 with rat 1 being the rat immunized with the RDSM peptide in the first round of immunizations) showed reactivity in ELISA with the corresponding peptide (data not shown). Sera from rat 4 and 7 also showed reactivity in FACS (Fig. 3, S4A and S4B).

### Peptide Array Reveals Specific Antibody Recognition

**Figure 4:**
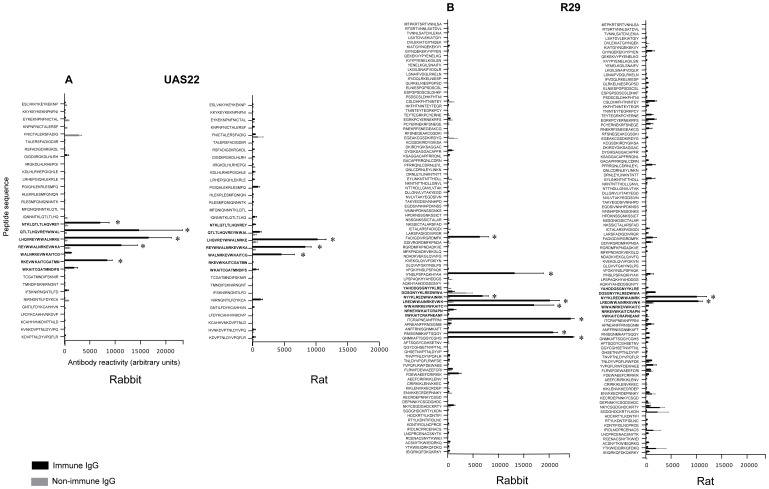
Peptide array mapping the rabbit and rat anti-DBL1α-RDSM IgG binding. The amino acid sequences are read from N- to C-terminal. The amino acids included in the RDSM peptide are shown in bold letters. Statistically significant changes comparing immune IgG and non-immune IgG are marked with an asterisk * P<0.05. The rat anti-DBL1α-RDSM IgG is from rat 1. **A.** DBL1α domain from UAS22. The rabbit/rat anti-DBL1α-RDSM IgG is shown in black and the non-immune IgG is shown in grey. **B.** DBL1α domain from R29. The rabbit/rat anti-DBL1α-RDSM IgG is shown in black and the non-immune IgG is shown in grey.

In order to map the antibody binding of the anti-DBL1α-RDSM IgG in detail, specific peptide arrays holding overlapping 15-mer peptides were produced as in [33]. The arrays hold five NTS-DBL1α domains from long-term cultivated parasites (FCR3S1.2_var1_, FCR3S1.2_var2_, R29, Palo Alto_varO_, 3D7_var4_) and shorter DBL1α tags from three Ugandan isolates (UAS22, UAS29 and UAS31) (see Table S1A and B). The peptide array confirmed a correlation between antibody recognition in the RDSM area of DBL1α in the array and antibody reactivity in FACS. Rat anti-DBL1α-RDSM IgG reacted on the microarray with peptides corresponding to the parasites that were positive in FACS (Fig. 4A, 4B and S5) while there was no reactivity with the peptides in the NTS-DBL1α sequences from the parasites that were negative in FACS. The rabbit anti-DBL1α-RDSM IgG also showed reactivity in the same area; however the recognition pattern was somewhat different (Fig. 4A, 4B and S6) reacting also with FCR3S1.2.

### Alanine Walking Identifies Essential Amino Acids for Antibody Binding

**Figure 5:**
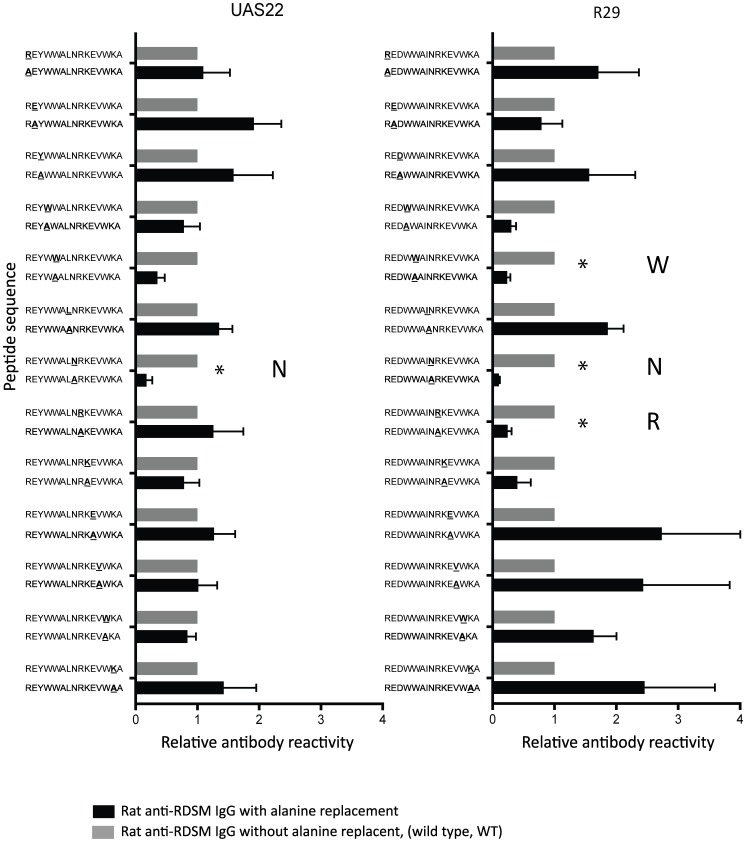
Alanine replacement peptide array of the DBL1α domains of parasites UAS22 and R29. Rat anti-DBL1α-RDSM IgG is depicted in black and reactivity to the wild type (WT) sequence is shown in grey as a reference. The reactivity is shown as a ratio between modified and WT sequence. Statistically significant changes compared to WT sequence are marked with an asterisk. * P<0.05.

In order to map the antibody binding and specificity in detail, alanine replacement peptide arrays were produced, i.e. peptide arrays with individual alanine point mutations covering the reacting RDSM area of five DBL1α sequences (FCR3S1.2_var1_, FCR3S1.2_var2_, R29, Palo Alto_varO_, UAS22; Table S1C). For the rat anti-DBL1α-RDSM IgG, the most important amino acid in the UAS22 sequence was an asparagine (N) in the middle of the motif sequence (REYWWAL**N**RKEVWKA) (Fig. 5). For the R29 sequence, two additional amino acids were important for binding of the rat anti-DBL1α-RDSM IgG, namely the arginine (R) in the motif sequence as well as one tryptophan (W) located adjacent to the motif (REYW**W**ALN**R**KEVWKA) (Fig. 5). The binding recognition profiles of the anti-DBL1α-RDSM rat sera number 4 and 7 were also analyzed with the alanine replacement arrays which showed a similar recognition pattern with an asparagine being important for antibody binding (REYWWAL**N**RKEVWKA) (Fig. S7). However other residues also seem central for antibody binding in the UAS22 sequence including tryptophan and leucin residues (REYW**WAL**NRKEVWKA) for both sera. In the R29 sequence, in addition to the centrally placed asparagine, tryptophans on both sides of the motif as well as a lysine (REY**W**WALNRKEV**WK**A) were important for antibody binding.

**Figure 6:**
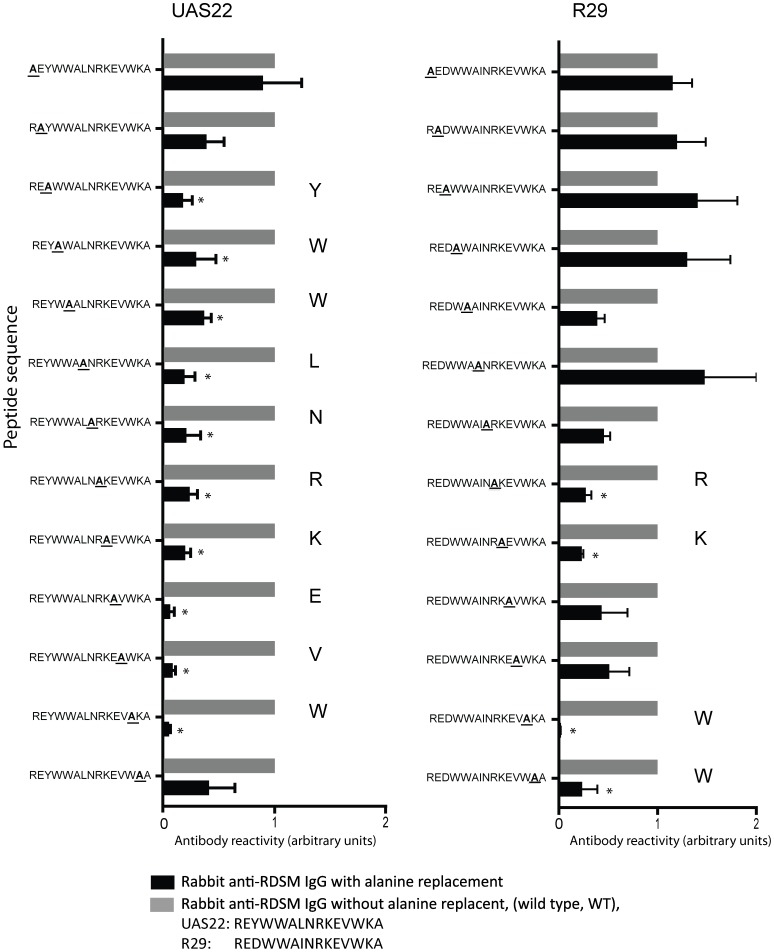
Alanine replacement peptide array of the DBL1α domains of parasites UAS22 and R29. Rabbit anti-DBL1α-RDSM IgG is depicted in black and reactivity to the WT sequence is shown in grey as a reference. The reactivity is shown as a ratio between modified and WT sequence. Statistically significant changes compared to WT sequence are marked with an asterisk. * P<0.05.

For rabbit anti-DBL1α-RDSM IgG the reactivity diminished in a number of peptide sequences from UAS22 and R29 (Fig. 6). However the most important amino acids seem to be located at the end of the UAS22 sequence, with amino acids glutamic acid, valine and tryptophan (EVW) being important for antibody binding (REYWWALNRK**EVW**KA). For the R29 sequence, alanine replacement of the tryptophan at the end of the sequence completely abolished the binding of the rabbit anti-DBL1α-RDSM IgG.

### Cross-reactivity with Different 15-mers of the RDSM Degenerate Motif Sequence

**Figure 7:**
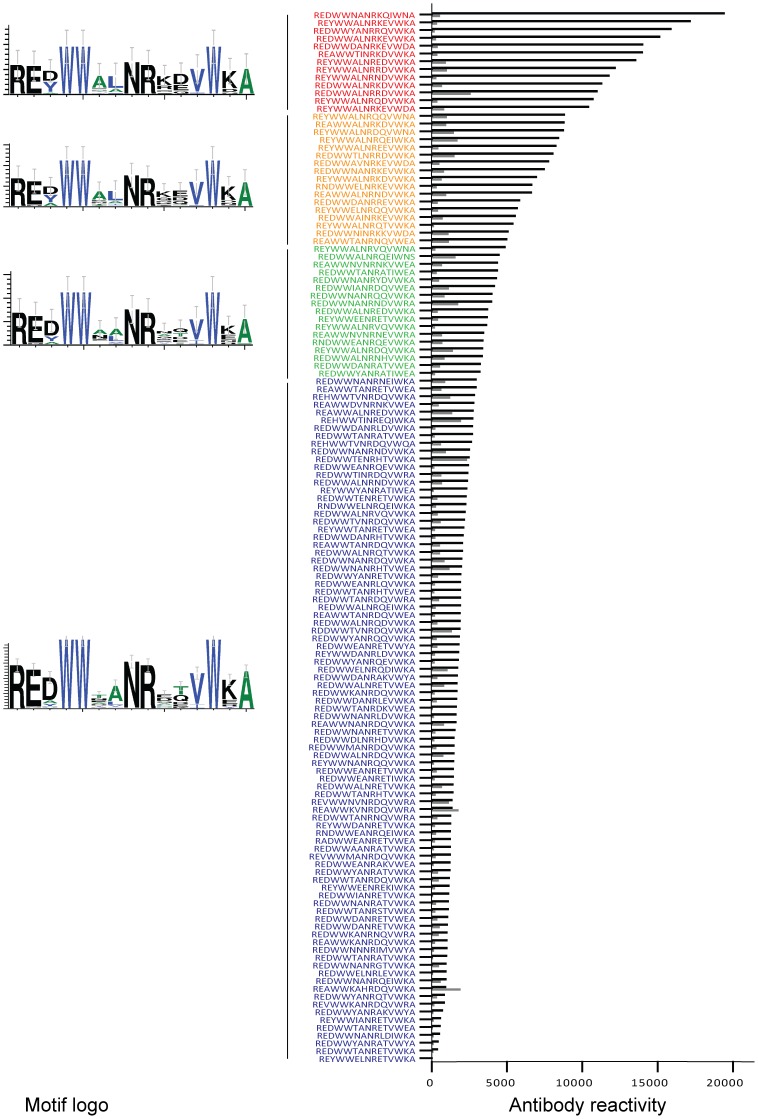
Antibody cross-reactivity to 135 unique RDSM peptide sequences. Rat anti-DBL1α-RDSM IgG recognition of 135 peptides covering the RDSM sequence motif. Degenerate motifs associated with different antibody recognition are shown to the left. Red letters indicates high reactivity, orange medium reactivity, green low reactivity and blue no reactivity.

The cross-reactive potential of the rat anti-DBL1α-RDSM IgG was explored using a third peptide array with 15-mer peptides of all identified unique sequences in the RDSM area from the Ugandan material (Table S1D) [30]. Of the 241 sequences included in the Ugandan study, 135 were unique sequences and 47 showed reactivity with the anti-DBL1α-RDSM IgG, albeit at different degrees. As seen in Fig. 7, high reactivity was seen with peptide sequences containing leucin, lysine and aspartic or glutamic acid (**AL**NR**KE**/**D**). This amino acid composition gradually diminished in conjunction with lower antibody recognition. In the sequences showing medium reactivity with the rat anti-DBL1α-RDSM IgG, aspartic acid was replaced by glutamic acid to some degree (**AL**NR**KE**). In the sequences with low or no reactivity, leucin was replaced by an alanine and the positively charged lysine was replaced by the negatively charged aspartic or glutamic acid (T**A**NR**E**/**D**). In addition, the aspartic or glutamic acid were replaced by threonine or glutamine (T**A**NR**ET**/**Q**). No correlation between the recognition patterns of the rat anti-DBL1α-RDSM IgG and the rabbit anti-DBL1α-RDSM IgG was found (Fig. S8).

## Discussion

It is not fully understood how protective immunity to *P. falciparum* malaria is acquired. Antibodies to the variant surface antigen PfEMP1 are part of this immunity but the exact location of the antibody binding sites in this multi-domain variant protein remains unknown. Herein we show that a linear sequence motif (ALNRKE) present in the subdomain 2 (SD2) of PfEMP1-DBL1α from a Ugandan isolate (UAS22) can induce surface reactive antibody responses. The anti-DBL1α-RDSM antibodies display live cell surface reactivity with seven out of 14 parasites tested, both laboratory strains and *in vitro* adapted clinical isolates of early generations, suggesting that the identified PfEMP1-DBL1α sequence motif is available at the pRBC surface. Similarly, dot blots performed with the rat anti-DBL1α-RDSM antibodies to recombinant proteins of NTS-DBL1α-domains from FCR3S1.2_var1_, FCR3S1.2_var2_, R29 and Palo Alto_varO_ showed reactivity with the R29 protein but not for the other included proteins that lack the motif (data not shown). This is in concordance with the FACS and the live cell IFA data. We further utilized a peptide array to map the binding properties of the elicited anti-DBL1α-RDSM antibodies and the amino acids WxxNRx were found essential for antibody binding. In complementary experiments using 135 degenerate RDSM peptide-sequences obtained from 93 Ugandan patient-isolates we found that antibody binding occurred predominantly when the amino acids WxLNRKE/D were present in the peptide. The data suggest that the identified epitope in SD2 could be a target for the induction of strain-transcending antibodies. These antibodies could be involved in opsonisation and phagocytosis but not in the disruption of rosettes since none of the obtained antibodies disrupted rosettes in any of the included parasite strains or clinical isolates (data not shown). However rosette disruption has been shown with antibodies to the loop of the flanking subdomain 3 (SD3) [34]. In a recent study, Ghumra *et al* [28] describe surface reactive antibodies with strain-transcending activity. Some of these antibodies did not have rosette inhibiting effect and one could speculate that the ALNRKE-sequence could be one of the epitopes important for the strain-transcending activity shown in the study. The RDSM motif is present in the homology block 5 (HB5) which is suggested to be frequently exposed on the surface of PfEMP1 [35]. HB5 is one of the most prevalent homology blocks and is present in nearly all DBL domains [35]. No correlation was found between the RDSM motif and the different PfEMP1 domain cassettes recently reported to be associated with severe malaria [36], [37], [38], [39].

The RDSM peptide that includes both the motif (ALNRKE) and surrounding amino acids (Table 1) was used to immunize both rabbits and rats, but only rats induced surface reactive antibodies. A peptide array with alanine point mutations in the RDSM sequence revealed that amino acids important for binding vary to some extent between different parasite sequences. Still an asparagine (N), an arginine (R) as well as a leucin (L) and a lysine (K) (A**LNRK**EVW) seem to be important for binding of the rat anti-DBL1α-RDSM antibodies. In contrast, for the rabbit anti-DBL1α-RDSM antibodies (that were negative in FACS) the most important amino acids seem to be glutamic acid (E), valine (V) and tryptophan (W) (ALNRK**EVW**), at the C-terminus of the sequence, just outside the RDSM motif. Indeed, the two amino acids (VW) in this area of the DBL1α-domain are conserved in a broad range of DBL1α sequences from different geographical locations. The two amino acids are situated just upstream of the PoLV3 motif [40] and are commonly used as an anchor when aligning DBL1α-domains. This pattern of conservation might be the reason why the rabbit anti-DBL1α-RDSM antibodies reacted with all tested parasite strains and isolates on dried monolayers of pRBC but not at the surface of the pRBC. On air-dried monolayers, the RBC membrane is disrupted and the antibodies are able to detect antigens inside the cell. One could speculate that the valine and tryptophan are exposed during the export of the PfEMP1 in the pRBC cytosol but are hidden upon exposure of the protein at the surface of the pRBC.

When the rat anti-DBL1α-RDSM IgG were assayed on a peptide array with 135 unique sequence-combinations of the RDSM peptide, high antibody recognition was shown in peptide sequences containing leucin, lysine and aspartic or glutamic acid as part of the motif (**AL**NR**KD**/**E**). In the sequences with no reactivity, leucin was replaced by an alanine and the positively charged lysine replaced by the negatively charged aspartic or glutamic acid (T**A**NR**E**/**D**). Interestingly, Dahlbäck *et al* have characterized a motif in the same location as the RDSM motif in the DBL3X domain of the pregnancy associated PfEMP1, VAR2CSA [41]. In placental isolates from Senegalese women, the EIEKD motif was associated with parasites from primi-gravidae and the motif GIEGE and G/EIERE with parasites from multi-gravidae. Comparing the three motifs there is a replacement from a lysine (EIE**K**D) in the motif associated with parasites from primi-gravidae to an arginine or glycine (EIE**R**E, GIE**G**E) in the motifs associated with parasites from multi-gravidae. This correlates with the findings herein. Preliminary data from our group indicate that there is reactivity to the ALNRKE-peptide with human sera from Cameroon (data not shown), however background reactivity with non-immune human IgG to the same area in the peptide array was also seen to various degrees (Albrecht *et al,* manuscript in preparation).

The sequence motifs used for peptide design in this study are all located in putative loops or α-helical semi-variable sequences of SD2 flanking subdomains 1 and 3 (SD1, SD3) the latter which are probably involved in mediating receptor binding of DBL1α [34], [42]. The hypervariable parts of the domain are unlikely to take part in any interaction due to the high sequence variability. The identified DBL1α-RDSM motif could be a linear epitope since it is both recognized on the peptide array as well as on the surface of the live pRBC which is similar to findings with *Plasmodium vivax* for the *P. vivax* Duffy binding protein (PvDBP) [43]. Mapping of naturally acquired antibodies, that prevented binding of PvDBP to reticulocytes, identified linear epitopes in the DBL domain of PvDBP which were mainly localized in SD2 [43]. The DBL domain of PvDBP (called RII-PvDBP) is similar in sequence and structure to other DBL domains of *Plasmodium* parasites including the DBL1α domain of PfEMP1. Batchelor *et al* show in a recent paper that PvDBP binds its receptor, DARC, via a receptor mediated dimerization [44]. The dimer interface is formed by the two SD2s of the DBL domains and the dimeric organization is crucial for receptor binding, creating a positively charged binding pocket for DARC. The authors suggest this model to be applicable to receptor recognition by other DBL domain-containing proteins such as PfEMP1 and PfEBA-175. Interestingly, the DBL1α-RDSM motif is situated in the SD2 of DBL1α and is close to the predicted binding pocket of PvDBP. However, as discussed before, none of the anti-DBL1α-RDSM antibodies showed rosette disrupting capacity. Preliminary data indicate that the epitopes important for rosetting are situated in SD3 and SD1 in the DBL1α-domain [34], [42].

The DBL1α-RDSM motif seems to be immunogenic since all seven rats immunized generated antibodies that showed surface reactivity with the live pRBC during the immunization schedule but the antibody reactivity was down-regulated upon repeated immunizations. Further, reactivity was seen for pre-immune sera of some animals in the peptide array. This may suggest that animals at least in-part are tolerized to the epitope and therefore diminish reactivity. A limited immunogenicity has also been seen with some HIV epitopes e.g. the monomeric CD4-binding site of gp120 as well as for epitopes of the surface glycoprotein haemagglutinin (HA) of influenza viruses [45].

In conclusion, this paper suggests that the identified PfEMP1-DBL1α-RDSM sequence is available at the parasitized erythrocyte surface of different clinical isolates and laboratory strains and that this motif can induce antibodies. In order to develop an anti-severe malaria vaccine much work is needed to identify the immunogens that can induce broad reactivity and functional antibodies that can confer protection by blocking sequestration and by opsonizing pRBCs for phagocytosis. Our results are a step forward to understand the basis of anti-plasmodial immune responses in order to identify the types of immunity required for a malaria vaccine that targets PfEMP1 at the surface of the pRBC.

## Materials and Methods

### Ethics Statement

Collection of human samples was conducted according to the principles expressed in the Declaration of Helsinki. Written informed consent was obtained from the patients or their legal guardians. The study was approved by Karolinska Institutet’s Regional Ethical Review Board (permission 03/095) and the Uganda National Council for Science and Technology (permission MV717). Animal immunizations were carried out by Agrisera (Umeå, Sweden) and were approved by Umeå Ethical Review Board (permissions A88-08 and A89-08). All animal work followed the Swedish Law and Regulations (1988∶534 and L55).

### Parasite Lines, Cultivation and Dominant Var Genes

A total of 14 *P. falciparum* isolates, strains or clones were used in this study. Of these, seven were clinical isolates: UAS22, UAS29, UAS31, UKS111, UAM15, UAM51, UKM62 collected in Uganda in 2002 and 2003 [30]. Patients were recruited in two locations in Uganda: at the district hospital in Apac, which is situated in a malaria holoendemic area [46] 250 km north of Kampala, and at the Mulago hospital, located in Kampala. In addition seven laboratory strains or clones from different geographical locations were used in the study including FCR3S1.2, FCR3S1.6, 3D7AH1, 3D7var4, HB3, NF54CSA and R29. For details of the included isolates and strains, see Tables 2 and 3. All 14 parasites were cultivated using standard methods developed by Trager and Jensen [47], [48] with the modifications that all *in vitro* adapted Ugandan isolates were cultivated in a gas mixture of 90% NO_2_, 5% O_2_ and 5% CO_2_ and by shaking the flasks as described [49]. Parasites were kept synchronous by intermittently treating the cultures with 5% sorbitol (w/v). The parasitemia was counted and the rosetting rate was determined by calculating the number of trophozoite pRBCs within rosettes, relative to the total number of trophozoite pRBCs present in the culture. A rosette was defined as at least two unparasitized RBCs bound to one pRBC. The rosetting rate was maintained with Ficoll enrichment as previously described [48]. FCR3S1.2 and R29 enrichment was carried out monthly with monoclonal antibodies (mAb) to the dominant PfEMP1 (IT4var60 and IT4var9 respectively) as described earlier [50], [51]. The 3D7var4 strain was a kind gift from Louise Joergensen, University of Copenhagen, Denmark and was similarly enriched on mAbs [52]. All experiments on FCR3S1.2, R29 and 3D7var4 were carried out within two weeks after mAb enrichment.

The dominantly expressed *var* genes for the clinical isolates UAS22, UAS29, UAS31, UAM15, UAM51 have been determined earlier [30]. In the paper by Normark *et al*, trophozoite stages parasites were used and *var* genes were amplified by reverse transcriptase-PCR with degenerate primers nDBLf (TKGCAGCMAAWTAYGARGX), nDBLr (KTCCACCAATCTTCYCT), α-AF (GCACGMAGTTTTGC) and α-BR (GCCCATTCSTCGAACCA), known to amplify ∼90% of *var* genes [30]. PCR products were cloned and forty-eight clones for each primer pair were subsequently sequenced using the MegaBace system. As described in [30], the sequence reads were base-called using phred, clustered using phrap and aligned using ClustalW. The number of generations between *var* gene sequencing and FACS/IFA is indicated in Table 2.

### Peptide Design

In order to investigate the involvement of the sequence motifs found in the DBL1α domains of PfEMP1 from the Ugandan isolates [30], we chose to design specific peptides, which were used in subsequent experiments. Please see Fig. S2 for an alignment of all included sequences. From the set of motifs we chose six motifs: four motifs that were associated with severe malaria and belonged to the groups defined in the Ugandan material as severe malaria 1 and 2 (S1S2), severe malaria 3/respiratory distress 5 (S3/R5, in this manuscript named RDSM in order to avoid confusion with the S3 domain of DBL1α [35]), high rosetting 1 and 2 (H1H2), high rosetting 3 (H3), and two motifs that were associated with mild disease and belonged to the low rosetting 1/mild malaria 4 (L1M4) and mild malaria 5 (M5) groups, respectively, see Fig. S1. These motifs were chosen because of predicted surface availability employing prediction program Phyre [53] and visualized by PyMOL (The PyMOL Molecular Graphics System, Version 1.3, Schrödinger, LLC). We further selected specific sequences from the pool that represent each degenerate motif based on the availability of *in vitro* adapted parasites predicted to express the specific motif. From the choice of specific sequences representing the motifs, we decided to extend the sequences in order to increase the likelihood of mimicking the native secondary structures that these motifs would have in the full-length protein. For this purpose, we used the Phyre webserver [53] to model the 3D structures of the DBL1α domains from which the sequences originated. Most sequences were part of α-helical structures: we therefore extended our selected sequences to include the full-length predicted α-helices. The resulting sequences were used as templates for the generation of peptides; the six different peptide sequences (16 to 25 amino acids long) are listed in Table 1.

### Tertiary Structure Modeling of NTS-DBL1α

The 3D structures of the NTS-DBL1α domains of R29 were modeled using the Phyre2 server with intensive settings [53]. The crystal structure of the NTS-DBL1α domain of Palo Alto_varO_ (Protein Data Bank accession code 2XUO) [42] was used as a template and had the highest sequence and secondary structure alignment score. Structural visualizations and high resolution images were made using PyMOL.

### Peptide Synthesis and Animal Immunization

An N-terminal cysteine was added to the peptides for conjugation to KLH (keyhole limpet hemacyanin) and for antibody affinity purification. The peptides were synthesized by Innovagen (Lund, Sweden). We pre-screened animal sera by Western blot using crude protein extracts of pRBCs and by IFA techniques on dried monolayers of pRBC and excluded those that showed pre-existing antibodies to the parasite or human red blood cell proteins to avoid background staining in subsequent assays. The selected animals (rats and rabbits) were immunized with peptides coupled to KLH, emulsified in Freund’s complete (first immunization) or incomplete adjuvant (second to fourth/fifth immunization). The amount of peptide was 200 µg the first and second immunizations and 100 µg the third, fourth and fifth immunizations for both rats and rabbits. Animal immunizations were carried out by Agrisera AB (Umeå, Sweden).

### Affinity Purification of Peptide Antibodies and Enzyme Linked-immunosorbent Assay (ELISA)

Affinity purification of serum antibodies on the respective peptide was done using UltraLink Iodoacetyl Matrix (Pierce, Thermo Scientific, Waltham, MA, USA) according to the manufacturer’s instructions. In summary, affinity columns were prepared by coupling 5 mg of each peptide to 2 ml of matrix for 45 min at room temperature (RT). After washes with five gel-bed volumes of coupling buffer, the matrix was blocked by incubation of L-cysteine-HCl for 45 min at RT. The sera were diluted 1∶2 (rabbit) or 1∶3 (rat) in PBS and were allowed to bind to the peptide matrix for 1 hour at RT. The column was washed with five gel-bed volumes of PBS (pH 7.4). The bound antibodies were eluted with 0.1 M glycine-HCl (pH 2.5) and immediately neutralized with 1 M Tris-HCl (pH 7.5). The antibody content of the eluted fractions was measured by a Nanodrop spectrophotometer (Thermo Scientific). The fractions containing antibodies were pooled. Antibodies were subsequently dialyzed against PBS pH 7.4 before they were stored in small aliquots at −20°C until used.

Specificities and titers of the antibodies were confirmed by ELISA; in short peptides were coated overnight at 4°C on MaxiSorp plates (Nunc, Roskilde, Denmark) at a concentration of 4 µg/ml. All sera were added and titrated by serial dilutions of 1∶121-1∶161051 in PBS with 0.05% Tween (TPBS). The antibodies were allowed to bind for two hours at RT and after three washes with TPBS, goat anti-rabbit total Ig and rabbit anti-rat total Ig, both conjugated to HRP (horseradish peroxidase) (DAKO, Glostrup, Denmark) were added at a concentrations of 1∶8000 and 1∶3500 respectively. After incubation for 30 min at RT and subsequent washes, the reaction was developed with EC Blue substrate system (Medicago AB, Uppsala, Sweden) and optical density was read at 650 nm.

### Immunofluorescence Assay on Dried pRBC and Live Parasite Immunofluorescence Assays

Air-dried monolayers of trophozoite-pRBC of the parasites FCR3S1.2, UAS22, UAS29, UKS111, R29 and 3D7AH1 were prepared as previously described [54]. The monolayers were blocked with 2% bovine serum albumin (BSA) in PBS and then incubated with anti-peptide serum at dilutions 1∶50, 1∶100, 1∶200, 1∶400, 1∶800 (rabbit sera) and 1∶25, 1∶50, 1∶100, 1∶200, 1∶400 (rat sera) and for affinity purified IgG 48, 24, 6 and 3 µg/ml, respectively. The dilutions were in PBS and the antibodies were allowed to bind for 60 min at RT. The slides were washed three times in PBS and primary antibodies were detected with an ALEXA488-conjugated goat anti-rat or goat anti-rabbit IgG (Molecular Probes, Invitrogen, Carlsbad, CA, USA), diluted to 1∶1000 for 60 min. All incubations were carried out at RT in a humid chamber. Slides were mounted with Vectashield anti-fading media containing DAPI for nuclear staining (Vector Laboratories, Inc., Burlingame, CA, USA). The monolayers were analyzed with 60x and 100x oil immersion lenses in a Nikon Eclipse 80i microscope (Nikon Corporation, Japan). Pictures were acquired with an Hamamatsu ORCA camera (Hamamatsu Photonics System, Hamamatsu City, Japan) and analyzed with ImageJ [55].

### Flow Cytometry Assay on Parasitized Erythrocytes and Live Cell Fluorescence Microscopy

The capacity of antibodies to bind to the surface of pRBC parasitized by trophozoite stage parasites (24–30 hours post merozoite invasion) was tested with flow cytometry as previously described [51]. Briefly, the pRBCs were washed in PBS with 2% FCS and then pre-incubated in the same medium for 30 min. Subsequently, the cells were incubated with either serum (final bleed or pre-bleed) in dilution 1∶5 or with affinity purified IgG at a concentration of 220 µg/ml for 30 min at RT. Non-immune rat/rabbit IgG (Jackson ImmunoResearch Laboratories, Inc, West Grove, PA, USA) were used as controls in the experiments with affinity purified IgG at identical concentration. Subsequent to the incubation with the primary antibody, the pRBCs were washed thrice with FCS/PBS. After a 30 min incubation with a goat anti-rat/rabbit IgG antibody coupled to ALEXA488 (dilution 1∶100), the pRBCs were washed thrice with FCS/PBS. Ethidium bromide was added at a final concentration of 2.5 µg/ml after the last wash and the pRBCs were resuspended in FCS/PBS after two additional washes. The cell acquisition was done using flow cytometry (FACSCalibur, BD Bioscience, Franklin Lakes, NJ USA) where 5000 pRBCs were counted. The analysis was performed using the FlowJo software (Tree Star, Inc. Ashland, Oregon, USA). All assays were done in duplicates. Reactivity of the pRBCs was scored as geometric mean fluorescence intensity as described previously [56]. In order to compare the different FACS acquisitions, the mean fluorescence of the pRBCs was calculated with the following formula, taking into account the background reactivity of unparasitized RBCs: (pRBC_immune_/pRBC_non-immune_) – (RBC_immune_/RBC_non-immune_). This formula is less sensitive to changes in the baseline voltage and thus less sensitive to changes in the set up of the FACS instrument.

### Peptide Array and Alanine Replacement Array

In order to map the antibody binding, specific peptide arrays containing overlapping peptides were produced. The peptides were bound chemoselectively to the microarray surface by coupling an active amine (from the peptide) to an epoxy-group (from the slide surface) by JPT Peptide Technologies, Berlin, Germany. The peptides were 15 amino acids long with an 11 amino acids overlap. Each slide held three identical subarrays.

Array number one (*Peptide array 1*) held peptides corresponding to five NTS-DBL1α-domains from the long-term cultivated parasites FCR3S1.2_var1_, FCR3S1.2_var2_, R29, Palo Alto_varO_, 3D7var4 and three shorter DBL1α sequences from the Ugandan isolates UAS22, UAS29 and UAS31 (Table S1A and B). To map the essential amino acids required for antibody binding, an alanine replacement array was produced in the same way (*Peptide array 2*). The included peptides and the alanine replacements are shown in Table S1C. Array number three (*Peptide array 3*) held peptides containing 135 peptides covering the RDSM motif area. These 135 sequences were obtained from the 93 isolates obtained from Ugandan children; a total of 241 sequences had been retrieved but 116 were duplicated in sequence as to the RDSM motif generating 135 unique sequences (Table S1D).

#### Assay procedure

The slides were incubated for 16 hours at 4°C in a humid chamber with rabbit- or rat affinity purified IgG (5 µg/ml) or total rat serum (1∶100) in PBS containing 3% of FCS and 0.5% of Tween (TPBS). Five washing steps followed each incubation step, two with TPBS, followed by three washing steps with distilled water. All the solutions were sterile filtered using a 0.22 µm filter. The slides were then incubated with a 1∶500 dilution of goat anti-rat- or goat anti-rabbit Cy5-conjugated secondary antibodies (Jackson ImmunoResearch) for two hours at RT in a humid chamber, followed by the five washing steps (see above). The microarrays slides were scanned at wavelength of 635 nm using a GenePix 4000B microarray scanner (Axon Instruments, Sunnyvale, CA, USA). The images were analyzed using GenePixPro 7.0 software in combination with the.GAL file provided by JPT. The mean of fluorescence intensity given by the difference between the foreground and the local background were used to measure the IgG response. The data represent the average of the three subarrays. Anova test and Bonferroni post test were applied to identify peptides that were differentially recognized by the immunized animals compared to their controls (pre-immune serum or purified non-immune IgG). The degenerate motifs were generated using the WebLogo software [57].

### Rosette Disruption Assay

Rosette disruption assays were performed on pRBC cultures of trophozoites 20–24 hours post invasion as described elsewhere [48]. Briefly, serum or IgG were added in dilutions 1∶5 and 1∶10 or 220 and 110 µg/ml in 40 µl of parasite culture, 5% hematocrit. After incubating the samples at RT for 30 minutes the parasites were stained with acridine orange and the rosettes were counted. For each sample, 25 fields, equivalent to approximately 3500–4000 RBCs, were counted. In parallel we also counted the rosetting rate for control samples treated with non-immune rat/rabbit serum or IgG at identical concentrations.

## Supporting Information

Figure S1 **Degenerate PfEMP1 sequence motifs.** Shown are the degenerated PfEMP1 motifs that were associated with severe malaria (S), mild malaria (M), high rosetting rate (H) or low rosetting rate (L) in clinical isolates from Uganda (Normark et al, PNAS 2007). Eight sequence motifs where chosen for immunization depending on their predicted surface availability: H1, H2, H3, S1, S2, S3, L1/M4, M5.
(PDF)

Figure S2 **Alignment of included sequences.** All DBL1α sequences from the Normark paper (Normark et al, PNAS 2007) are aligned to show how degenerate the RDSM motif is.
(PDF)

Figure S3 **Indirect IFA on air-dried monolayers of the clinical isolate UAS22.** Specific donut pattern with rabbit anti-DBL1α-RDSM antibodies (green) on a panel of parasite strains and isolates. The parasite nucleus is stained with DAPI (blue).
(PDF)

Figure S4 **Peptide array mapping rat anti-DBL1α-RDSM antibody binding. A. Serum from rat 4.** DBL1α domains from UAS22 and R29. The rat 4 anti-DBL1α-RDSM serum is shown in black and pre-immune serum is shown in grey. The amino acid sequences are read from N- to C-terminal. The amino acids included in the RDSM peptide are shown in bold letters. **B.** **Serum from rat 7.** DBL1α domains from UAS22 and R29. The rat anti-DBL1α-RDSM serum is shown in black and the pre-immune serum is shown in grey. The amino acid sequences are read from N- to C-terminal. The amino acids included in the RDSM peptide are shown in bold letters.
(PDF)

Figure S5 **Peptide array mapping the binding of rat anti-DBL1α-RDSM IgG.** The DBL1α domains assayed are from FCR3S1.2_var2,_ 3D7var4 and Palo Alto_varO_. The amino acid sequences are read from N- to C-terminal. The rat anti-DBL1α-RDSM IgG is shown in black and the non-immune rat IgG is shown in grey. Statistically significant changes comparing rat anti-DBL1α-RDSM IgG and non-immune rat IgG are marked with an asterisk. * P<0.05.
(PDF)

Figure S6 **Peptide array mapping the binding of rabbit anti-DBL1α-RDSM IgG.** The DBL1α domains assayed are from FCR3S1.2_var2,_ 3D7var4 and Palo Alto_varO_. The amino acid sequences are read from N- to C-terminal. The rabbit anti-DBL1α-RDSM IgG is shown in black and the non-immune rabbit IgG is shown in grey. Statistically significant changes comparing rabbit anti-DBL1α-RDSM IgG and non-immune rabbit IgG are marked with an asterisk. * P<0.05.
(PDF)

Figure S7 **Alanine replacement peptide array of the DBL1α domains of parasites UAS22 and R29.** Sera from rat 4 and 7 (final bleed) were run on the array. Rat 4 is shown in black and rat 7 in grey. The reactivity is shown as a ratio between modified and wild type sequence. Statistically significant changes comparing pre-immune and immune sera are marked with an asterisk, * P<0.05, **P<0.001.
(PDF)

Figure S8 **Reactivity to 135 unique RDSM peptide sequence.** Rabbit and rat anti-DBL1α-RDSM IgG recognition of 135 peptides covering the RDSM sequence motif. The peptide sequences are shown in order of reactivity to rat anti-DBL1α-RDSM IgG.
(PDF)

Table S1 **Peptide sequences included in the microarrays.**
(DOC)
