# Supplementary material for: A Sequence in Subdomain 2 of DBL1α of Plasmodium falciparum Erythrocyte Membrane Protein 1 Induces Strain Transcending Antibodies
Source: PLoS One. 2013 Jan 15;8(1):e52679. doi: 10.1371/journal.pone.0052679 (PMC3546040; doi:10.1371/journal.pone.0052679)
Supplement: Figure S1 — Degenerate PfEMP1 sequence motifs. Shown are the degenerated PfEMP1 motifs that were associated with severe malaria (S), mild malaria (M), high rosetting rate (H) or low rosetting rate (L) in clinical isolates from Uganda (Normark et al, PNAS 2007). Eight sequence motifs where chosen for immunization depending on their predicted surface availability: H1, H2, H3, S1, S2, S3, L1/M4, M5. (PDF) [file pone.0052679.s001.pdf]

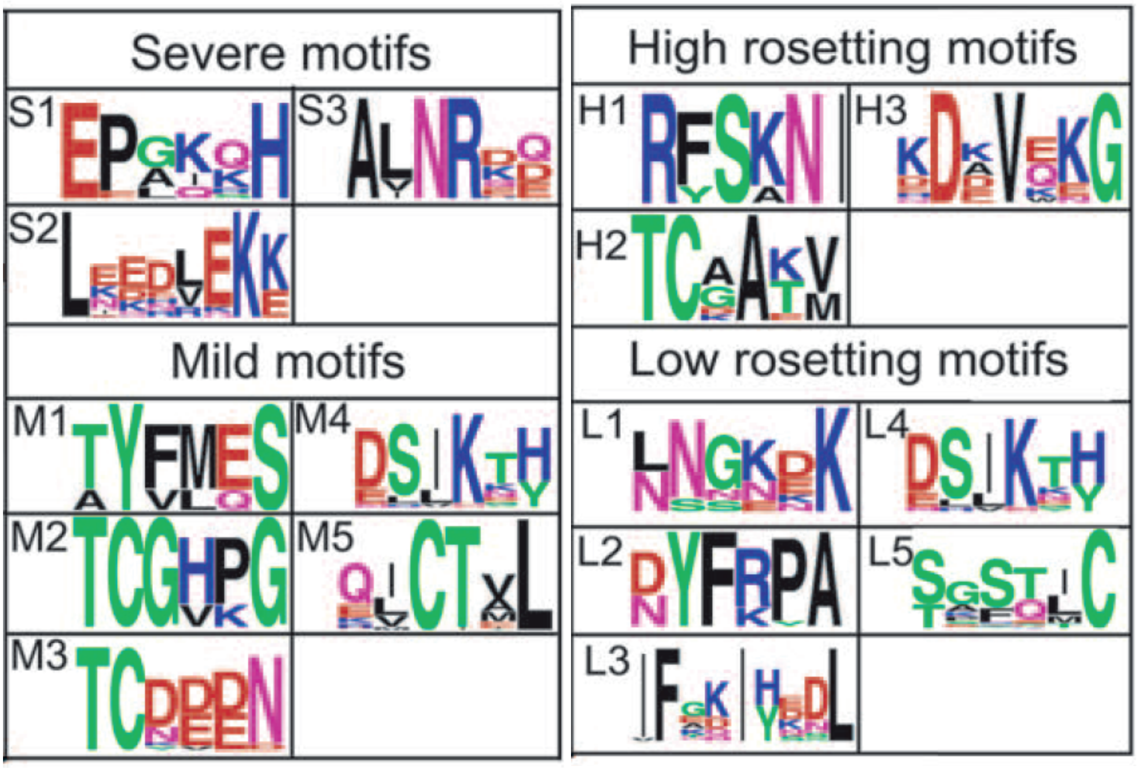
Severe motifs
High rosetting motifs
S1
S3
H1
H3
S2
H2
Mild motifs
Low rosetting motifs
M1
M4
L1
L4
M2
M5
L2
L5
M3
L3
