# Supplementary figures and images for: A Sequence in Subdomain 2 of DBL1α of Plasmodium falciparum Erythrocyte Membrane Protein 1 Induces Strain Transcending Antibodies
Source: PLoS One. 2013 Jan 15;8(1):e52679. doi: 10.1371/journal.pone.0052679 (PMC3546040; doi:10.1371/journal.pone.0052679)

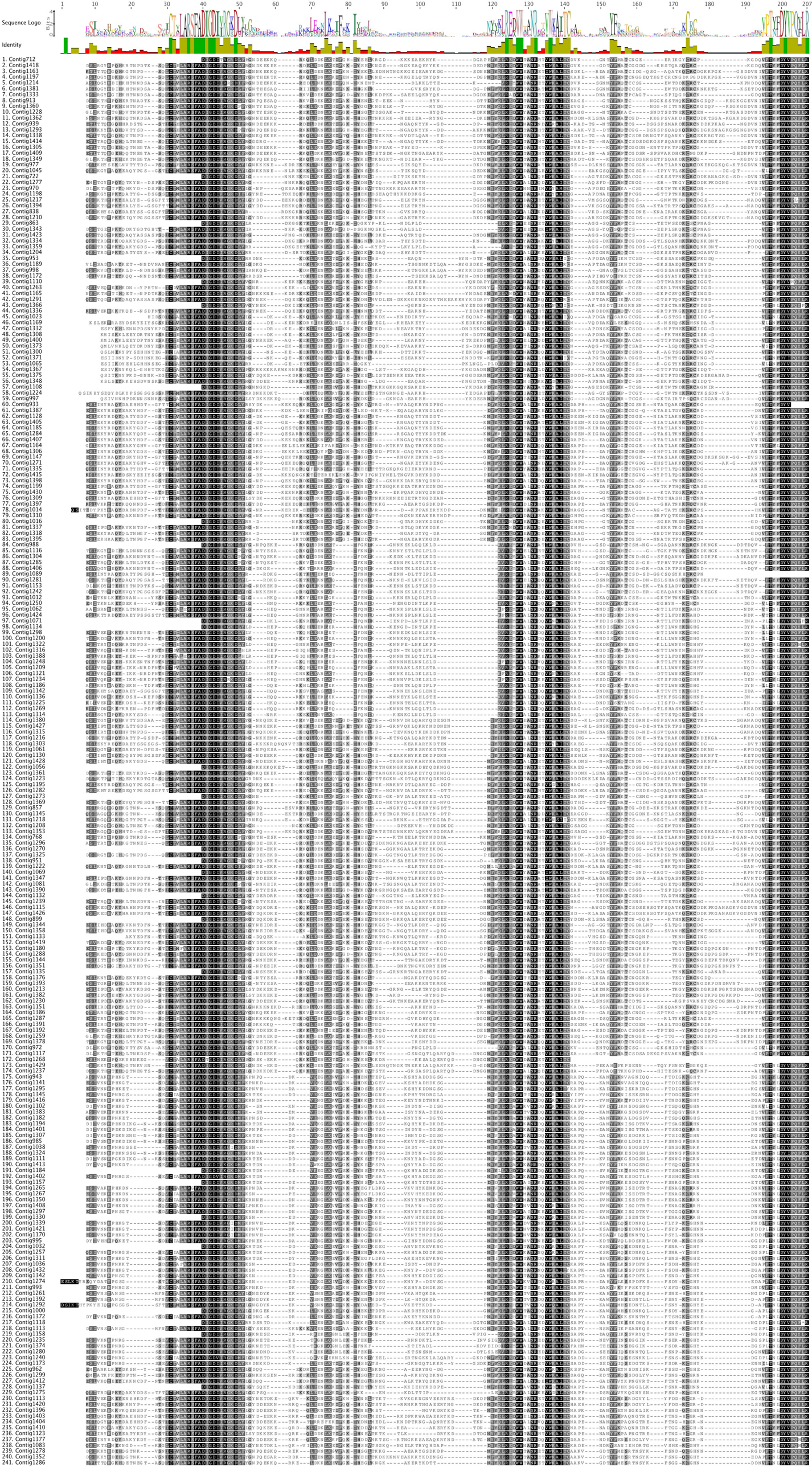

Supplement: Figure S2 — Alignment of included sequences. All DBL1α sequences from the Normark paper (Normark et al, PNAS 2007) are aligned to show how degenerate the RDSM motif is. (PDF) [file pone.0052679.s002.pdf]

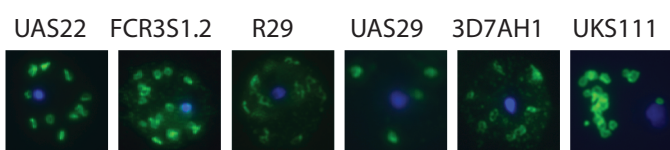

Rabbit anti-RDSM IgG

Supplement: Figure S3 — Indirect IFA on air-dried monolayers of the clinical isolate UAS22. Specific donut pattern with rabbit anti-DBL1α-RDSM antibodies (green) on a panel of parasite strains and isolates. The parasite nucleus is stained with DAPI (blue). (PDF) [file pone.0052679.s003.pdf]

UAS22

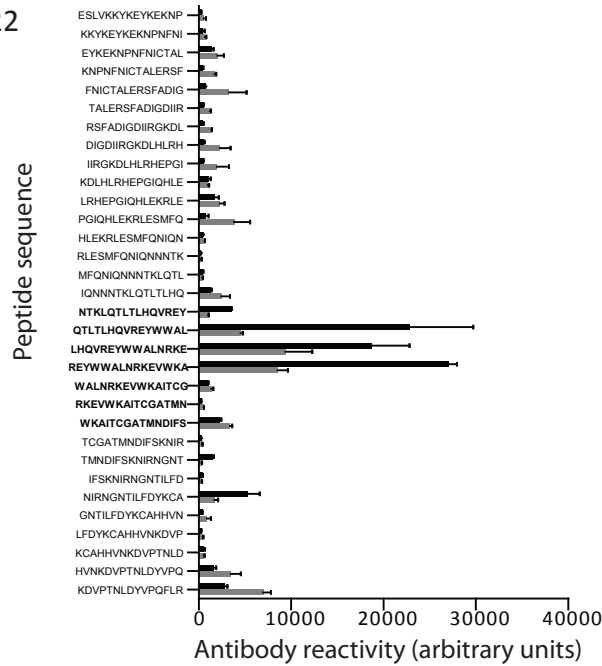

R29

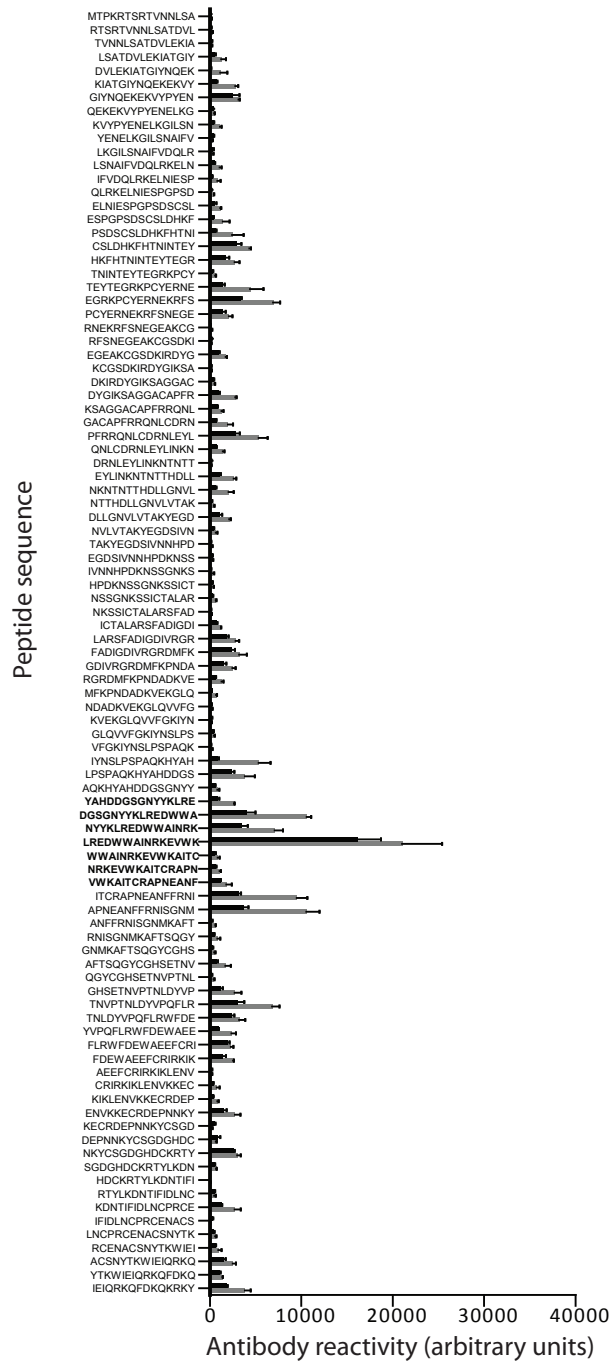

Supplement: Figure S4 — Peptide array mapping rat anti-DBL1α-RDSM antibody binding. A. Serum from rat 4. DBL1α domains from UAS22 and R29. The rat 4 anti-DBL1α-RDSM serum is shown in black and pre-immune serum is shown in grey. The amino acid sequences are read from N- to C-terminal. The amino acids included in the RDSM peptide are shown in bold letters. B. Serum from rat 7. DBL1α domains from UAS22 and R29. The rat anti-DBL1α-RDSM serum is shown in black and the pre-immune serum is shown in grey. The amino acid sequences are read from N- to C-terminal. The amino acids included in the RDSM peptide are shown in bold letters. (PDF) [file pone.0052679.s004.pdf]

■ Rat anti-RDSM IgG  
■ Rat non-immune IgG

FCR3S1.2 var2

3D7var4

Palo Alto varO

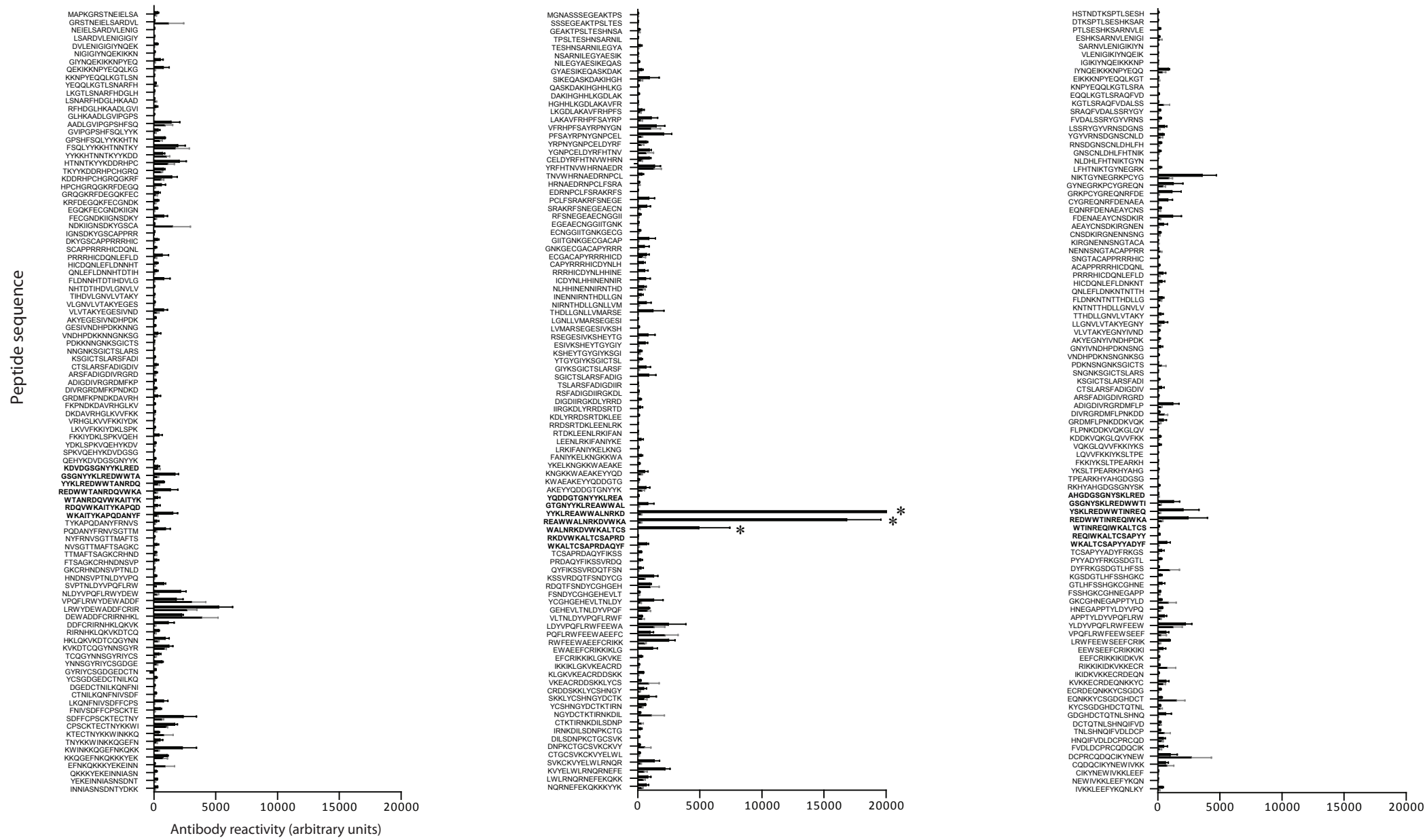

Supplement: Figure S5 — Peptide array mapping the binding of rat anti-DBL1α-RDSM IgG. The DBL1α domains assayed are from FCR3S1.2var2, 3D7var4 and Palo AltovarO. The amino acid sequences are read from N- to C-terminal. The rat anti-DBL1α-RDSM IgG is shown in black and the non-immune rat IgG is shown in grey. Statistically significant changes comparing rat anti-DBL1α-RDSM IgG and non-immune rat IgG are marked with an asterisk. * P<0.05. (PDF) [file pone.0052679.s005.pdf]

■ Rabbit non-immune IgG

Palo Alto varO

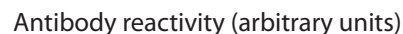

Supplement: Figure S6 — Peptide array mapping the binding of rabbit anti-DBL1α-RDSM IgG. The DBL1α domains assayed are from FCR3S1.2var2, 3D7var4 and Palo AltovarO. The amino acid sequences are read from N- to C-terminal. The rabbit anti-DBL1α-RDSM IgG is shown in black and the non-immune rabbit IgG is shown in grey. Statistically significant changes comparing rabbit anti-DBL1α-RDSM IgG and non-immune rabbit IgG are marked with an asterisk. * P<0.05. (PDF) [file pone.0052679.s006.pdf]

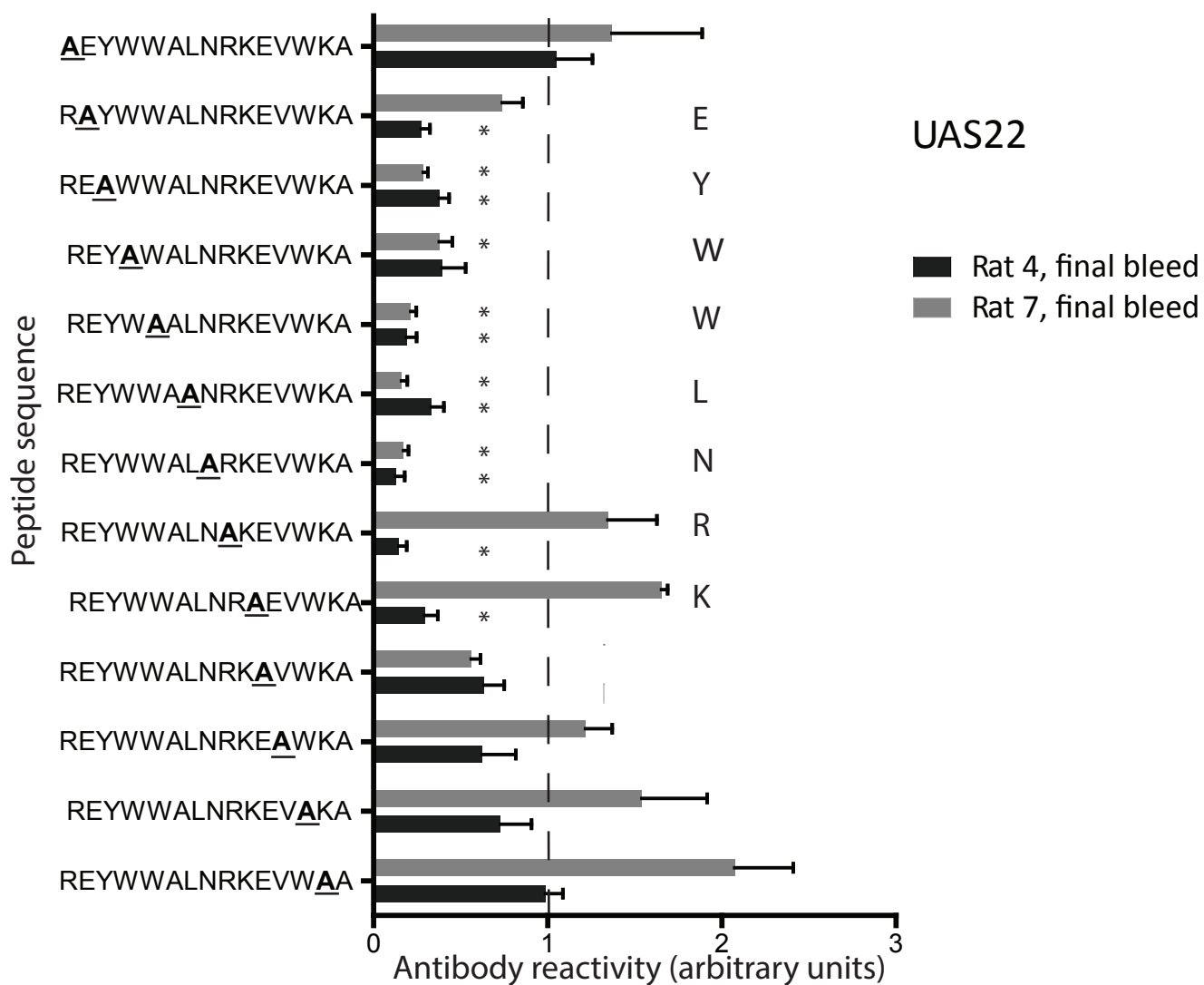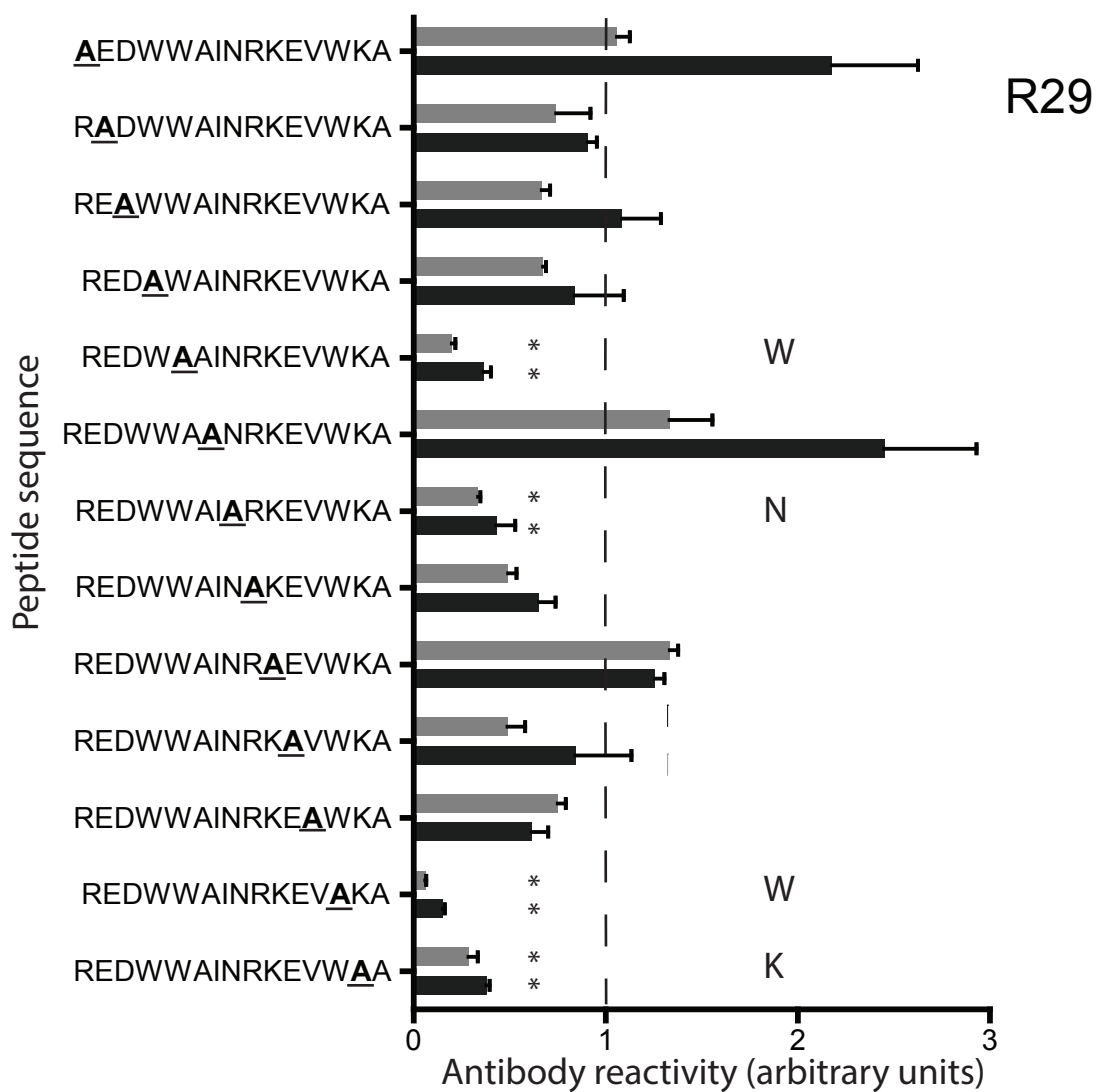

Supplement: Figure S7 — Alanine replacement peptide array of the DBL1α domains of parasites UAS22 and R29. Sera from rat 4 and 7 (final bleed) were run on the array. Rat 4 is shown in black and rat 7 in grey. The reactivity is shown as a ratio between modified and wild type sequence. Statistically significant changes comparing pre-immune and immune sera are marked with an asterisk, * P<0.05, **P<0.001. (PDF) [file pone.0052679.s007.pdf]

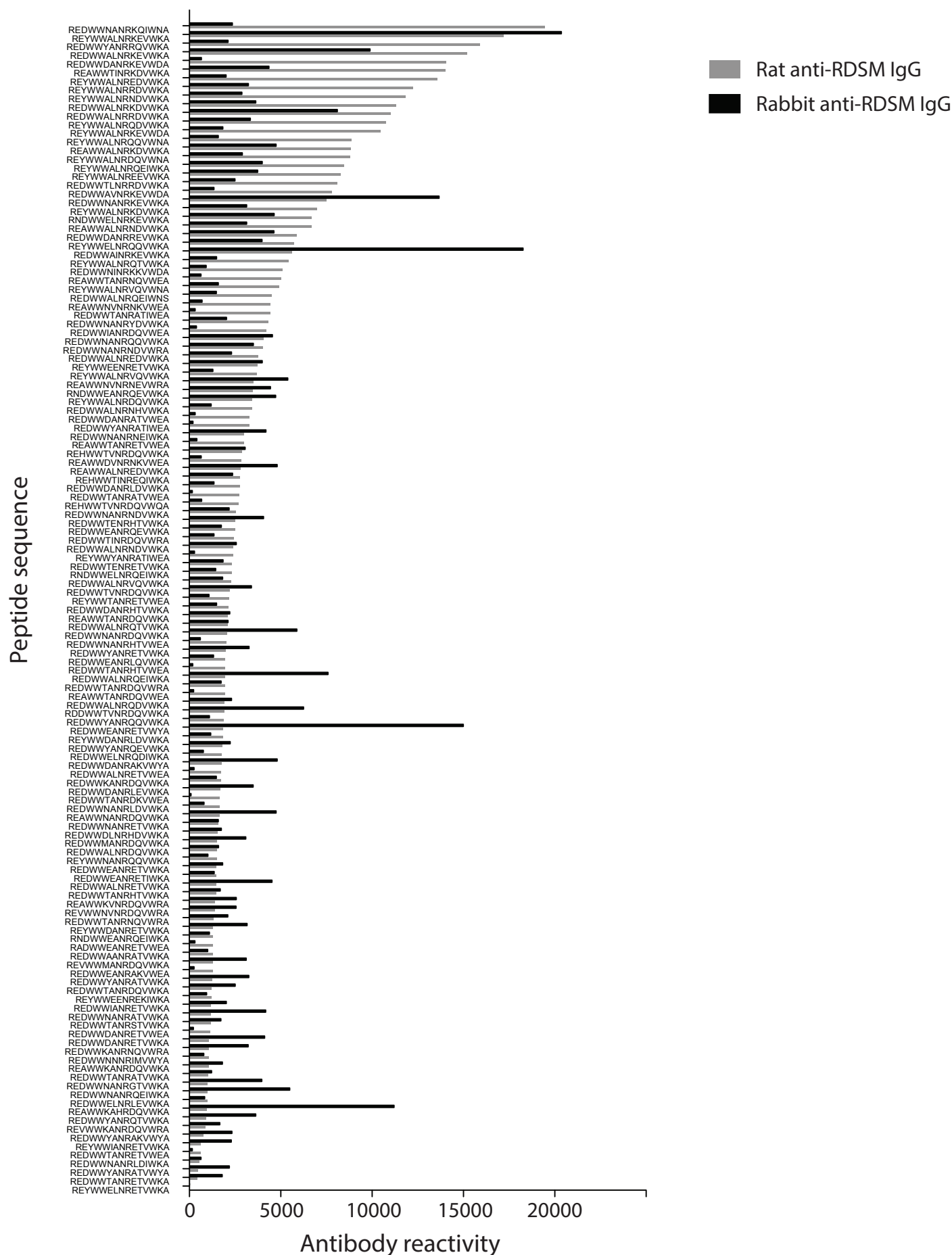

Supplement: Figure S8 — Reactivity to 135 unique RDSM peptide sequence. Rabbit and rat anti-DBL1α-RDSM IgG recognition of 135 peptides covering the RDSM sequence motif. The peptide sequences are shown in order of reactivity to rat anti-DBL1α-RDSM IgG. (PDF) [file pone.0052679.s008.pdf]
