# Supplementary material for: A Sequence in Subdomain 2 of DBL1α of Plasmodium falciparum Erythrocyte Membrane Protein 1 Induces Strain Transcending Antibodies
Source: PLoS One. 2013 Jan 15;8(1):e52679. doi: 10.1371/journal.pone.0052679 (PMC3546040; doi:10.1371/journal.pone.0052679)
Supplement: Table S1 — Peptide sequences included in the microarrays. (DOC) [file pone.0052679.s009.doc]

**Table S1.**

**A. Protein sequences on the peptide microarray**

| Parasite | Protein ID | Protein size on the array (aa) | Number of peptides (15aa) |
| --- | --- | --- | --- |
| FCR3S1.2 | var1 NTS-DBL1α | 415 | 101 |
| FCR3S1.2 | var2 NTS-DBL1α-CIDR1α | 782 | 193 |
| R29 | NTS-DBL1α | 391 | 95 |
| Palo Alto | varO NTS-DBL1α | 398 | 97 |
| 3D7 | var4 NTS-DBL1α | 399 | 97 |
| UAS22 | Contig 1388 DBL1α tag | 138 | 32 |
| UAS29 | Contig 1413 DBL1α tag | 140 | 33 |
| UAS31 | Contig 1429 DBL1α | 162 | 38 |

**B. Protein sequences included on the peptide array no 1:**

**DBL1 protein sequences**

# *FCR3S1.2 var1 NTS-DBL1α*

MATSGGSGGTQDEDAKHVLDEFGQKVHDEVHGEAKNYVSELKGSLSLASILGETAFTVKSMQTESKYTELI

EANSKRNPCKKDGKGNDVDRFSVKEQAGYDNKKMKCSNGDACAPFRRLHLCNKNFPNMNSYDSSKAKHDLLAEVCMAAKYEGESIKTHYPKYDSKYPGSDFPMCTMLARSFADIGDIIRGRDLYLGNKKKKQNGKETEREKLEQKLKEIFKKIHDNLKDKEAQKRYNGDEDPNFYKLREDWWTANRETVWGAMTCSKELDASSYFRATCSDTGQGPSQTHNKCRCDKDKGANAGKPKAGDGDVTIVPTYFDYVPQYLRWFEEWAEDFCRKKKKKLENLEKQCRGKDKSDEYRYCSRNGYDCEQTISRKGKVRMGKGCTDCFFACHSYENWIDNQRKQFDKQKKY

# *FCR3S1.2 var2 (IT4var60) NTS-DBL1α-CIDR1α*

MAPKGRSTNEIELSARDVLENIGIGIYNQEKIKKNPYEQQLKGTLSNARFHDGLHKAADLGVIPGPSHFSQ

LYYKKHTNNTKYYKDDRHPCHGRQGKRFDEGQKFECGNDKIIGNSDKYGSCAPPRRRHICDQNLEFLDNNHTDTIHDVLGNVLVTAKYEGESIVNDHPDKKNNGNKSGICTSLARSFADIGDIVRGRDMFKPNDKDAVRHGL

KVVFKKIYDKLSPKVQEHYKDVDGSGNYYKLREDWWTANRDQVWKAITYKAPQDANYFRNVSGTTMAFTSAGKCRHNDNSVPTNLDYVPQFLRWYDEWADDFCRIRNHKLQKVKDTCQGYNNSGYRIYCSGDGEDCTNILKQNFNIVSDFFCPSCKTECTNYKKWINKKQGEFNKQKKKYEKEINNIASNSDNTYDKKVYKTLKSMYPLDTKFVATLKEAPFCNNNNVDGIIDFNKPDDTFSSSTYCDSCPAFGVICENGTCTKVNEDTCSKMNVQVPKIITNKEDPTNIGILVSDDRVNVIPNELENGCKNTGIFKGIRKDEWSCKYLCNLDVCDLSHNKNNTHIDKRISIRVLFKRWLEYFLKDYSKLKKKLNSCTNNGKESICINECKKKCECVGKWAEEKRKEWEKVRKRFFNQYNVDDSLKSYEVKTFVNGNVDRSDIKNALNEGENLEALQDSDECIKPHNSKKDTCVKNDVVNILINRLKKKIDDCKIQHDNRTNQICCDELPESKEDNEDEEEEGEKKKNSKHLEETKEKKELDDNNFLDLCNNVKKYIEDNNKQISIQHK

*R29 NTS-DBL1α*

MTPKRTSRTVNNLSATDVLEKIATGIYNQEKEKVYPYENELKGILSNAIFVDQLRKELNIESPGPSDSCSLDHKFHTNINTEYTEGRKPCYERNEKRFSNEGEAKCGSDKIRDYGIKSAGGACAPFRRQNLCDRNLEYLINKNTNTTHDLLGNVLVTAKYEGDSIVNNHPDKNSSGNKSSICTALARSFADIGDIVRGRDMFKPNDADKVEKGLQVVFGKIYNSLPSPAQKHYAHDDGSGNYYKLREDWWAINRKEVWKAITCRAPNEANFFRNISGNMKAFTSQGYCGHSETNVPTNLDYVPQFLRWFDEWAEEFCRIRKIKLENVKKECRDEPNNKYCSGDGHDCKRTYLKDNTIFIDLNCPRCENACSNYTKWIEIQRKQFDKQKRKY

*varO NTS-DBL1α*

MGSSHSTNDTKSPTLSESHKSARNVLENIGIKIYNQEIKKKNPYEQQLKGTLSRAQFVDALSSRYGYVRN

SDGNSCNLDHLFHTNIKTGYNEGRKPCYGREQNRFDENAEAYCNSDKIRGNENNSNGTACAPPRRRHICD

QNLEFLDNKNTNTTHDLLGNVLVTAKYEGNYIVNDHPDKNSNGNKSGICTSLARSFADIGDIVRGRDMFL

PNKDDKVQKGLQVVFKKIYKSLTPEARKHYAHGDGSGNYSKLREDWWTINREQIWKALTCSAPYYADYFR

KGSDGTLHFSSHGKCGHNEGAPPTYLDYVPQFLRWFEEWSEEFCRIKKIKIDKVKKECRDEQNKKYCSGD

GHDCTQTNLSHNQIFVDLDCPRCQDQCIKYNEWIVKKLEEFYKQNLKY

*3D7var4 NTS-DBL1α*

MGNASSSEGEAKTPSLTESHNSARNILEGYAESIKEQASKDAKIHGHHLKGDLAKAVFRHPFSAYRPNYG

NPCELDYRFHTNVWHRNAEDRNPCLFSRAKRFSNEGEAECNGGIITGNKGECGACAPYRRRHICDYNLHH

INENNIRNTHDLLGNLLVMARSEGESIVKSHEYTGYGIYKSGICTSLARSFADIGDIIRGKDLYRRDSRT

DKLEENLRKIFANIYKELKNGKKWAEAKEYYQDDGTGNYYKLREAWWALNRKDVWKALTCSAPRDAQYFI

KSSVRDQTFSNDYCGHGEHEVLTNLDYVPQFLRWFEEWAEEFCRIKKIKLGKVKEACRDDSKKLYCSHNG

YDCTKTIRNKDILSDNPKCTGCSVKCKVYELWLRNQRNEFEKQKKKYYK

*UAS22 C1388 DBL1α*

ESLVKKYKEYKEKNPNFNICTALERSFADIGDIIRGKDLHLRHEPGIQHLEKRLESMFQN

IQNNNTKLQTLTLHQVREYWWALNRKEVWKAITCGATMNDIFSKNIRNGNTILFDYKCAH

HVNKDVPTNLDYVPQFLR

*UAS29 C1429 DBL1α*

ESLIHYHEQYQNKYGDSQLCTMLARSFADIGDIVRGKDLYRGNDEEKKQRDKLDKNFKKY

FQQIHDDVMSTKGRHGVKARYKADKNNNFFQLREDWWEANRETVWEALTCKADNSNRYFR

QTCNDNESLSHASHKCRCRSKNFKEETDQVPTYFDYVPQYLR

*UAS31 C1413 DBL1α*

DYIVQNHPNKDTSEVCTALARSFADIGDIVKGKDMFKRTDNDEVWKGLRAVFGKIYKSLP

SPAQNYYADDGSGNYYKLREAWWKAHRDQVWKAITCKAPPKVDYFIKNSDGSRGFTSQGQ

CGRNEINVPTNLDYVPQFLR

**C. Peptide array 2: Alanine replacement of included DBL1α domains.**

*FCR3S1.2 var 1*

**A**EDWWTANRETVWGA

R**A**DWWTANRETVWGA

RE**A**WWTANRETVWGA

RED**A**WTANRETVWGA

REDW**A**TANRETVWGA

REDWW**A**ANRETVWGA

REDWWTA**A**RETVWGA

REDWWTAN**A**ETVWGA

REDWWTANR**A**TVWGA

REDWWTANRE**A**VWGA

REDWWTANRET**A**WGA

REDWWTANRETV**A**GA

REDWWTANRETVW**A**A

REDWWTANRETVWGA

*FCR3S1.2 var 2*

**A**EDWWTANRDQVWKA

R**A**DWWTANRDQVWKA

RE**A**WWTANRDQVWKA

RED**A**WTANRDQVWKA

REDW**A**TANRDQVWKA

REDWW**A**ANRDQVWKA

REDWWTA**A**RDQVWKA

REDWWTAN**A**DQVWKA

REDWWTANR**A**QVWKA

REDWWTANRD**A**VWKA

REDWWTANRDQ**A**WKA

REDWWTANRDQV**A**KA

REDWWTANRDQVW**A**A

REDWWTANRDQVWKA

*R29*

**A**EDWWAINRKEVWKA

R**A**DWWAINRKEVWKA

RE**A**WWAINRKEVWKA

RED**A**WAINRKEVWKA

REDW**A**AINRKEVWKA

REDWWA**A**NRKEVWKA

REDWWAI**A**RKEVWKA

REDWWAIN**A**KEVWKA

REDWWAINR**A**EVWKA

REDWWAINRK**A**VWKA

REDWWAINRKE**A**WKA

REDWWAINRKEV**A**KA

REDWWAINRKEVW**A**A

REDWWAINRKEVWKA

*UAS22 C1388*

**A**EYWWALNRKEVWKA

R**A**YWWALNRKEVWKA

RE**A**WWALNRKEVWKA

REY**A**WALNRKEVWKA

REYW**A**ALNRKEVWKA

REYWWA**A**NRKEVWKA

REYWWAL**A**RKEVWKA

REYWWALN**A**KEVWKA

REYWWALNR**A**EVWKA

REYWWALNRK**A**VWKA

REYWWALNRKE**A**WKA

REYWWALNRKEV**A**KA

REYWWALNRKEVW**A**A

REYWWALNRKEVWKA

*varO*

**A**EDWWTINREQIWKA

R**A**DWWTINREQIWKA

RE**A**WWTINREQIWKA

RED**A**WTINREQIWKA

REDW**A**TINREQIWKA

REDWW**A**INREQIWKA

REDWWT**A**NREQIWKA

REDWWTI**A**REQIWKA

REDWWTIN**A**EQIWKA

REDWWTINR**A**QIWKA

REDWWTINRE**A**IWKA

REDWWTINREQ**A**WKA

REDWWTINREQI**A**KA

REDWWTINREQIW**A**A

REDWWTINREQIWKA

**D. Peptide sequences included in the peptide array no 3, containing 135 unique RDSM sequences.**

Contig1000 REDWWTANRDQVWKA

Contig1012 REYWWDANRLDVWKA

Contig1014 REDWWEANRAKVWEA

Contig1016 RNDWWEANRQEVWKA

Contig1023 RNDWWELNRQEIWKA

Contig1032 REDWWTANRNQVWRA

Contig1038 RDDWWTVNRDQVWKA

Contig1045 REDWWDANRLEVWKA

Contig1061 REDWWYANRETVWKA

Contig1062 REYWWALNRDQVWKA

Contig1069 REDWWYANRATIWEA

Contig1071 REYWWALNRDQVWNA

Contig1083 REDWWNANRKEVWKA

Contig1102 REAWWKVNRDQVWRA

Contig1108 REDWWYANRATVWYA

Contig1110 REDWWNANRYDVWKA

Contig1111 REAWWNANRDQVWKA

Contig1116 REYWWELNRETVWKA

Contig1117 REDWWTANRSTVWKA

Contig1118 REAWWALNREDVWKA

Contig1130 REDWWNANRETVWKA

Contig1133 REDWWDANRREVWKA

Contig1136 REYWWALNRQQVWNA

Contig1137 REDWWAVNRKEVWDA

Contig1147 REDWWTENRETVWKA

Contig1151 REDWWYANRQTVWKA

Contig1153 REYWWIANRETVWKA

Contig1157 REDWWAINRKEVWKA

Contig1158 REAWWALNRNDVWKA

Contig1163 REDWWEANRETIWKA

Contig1164 REDWWYANRQQVWKA

Contig1165 RNDWWELNRKEVWKA

Contig1169 REDWWNANRNEIWKA

Contig1170 REAWWNVNRNKVWEA

Contig1172 REDWWTANRATVWKA

Contig1173 REDWWDANRETVWKA

Contig1182 REAWWTANRDQVWKA

Contig1184 REDWWKANRDQVWKA

Contig1186 REYWWALNREEVWKA

Contig1189 REDWWALNRRDVWKA

Contig1195 REDWWDANRKEVWDA

Contig1197 REDWWDANRETVWEA

Contig1198 REDWWDANRAKVWYA

Contig1204 REDWWALNRKEVWKA

Contig1208 REDWWALNRQTVWKA

Contig1209 REYWWALNRVQVWKA

Contig1210 REDWWNANRHTVWEA

Contig1214 REYWWDANRETVWKA

Contig1216 REDWWALNRETVWKA

Contig1218 REDWWALNRQEIWNS

Contig1222 REAWWTANRETVWEA

Contig1224 REDWWIANRETVWKA

Contig1230 REDWWAANRATVWKA

Contig1234 REYWWALNRKEVWKA

Contig1235 REDWWALNREDVWKA

Contig1242 REYWWNANRQQVWKA

Contig1257 REDWWKANRNQVWRA

Contig1259 REDWWNANRGTVWKA

Contig1261 REHWWTVNRDQVWKA

Contig1265 REDWWMANRDQVWKA

Contig1267 REVWWMANRDQVWKA

Contig1271 REDWWALNRNDVWKA

Contig1273 REDWWNANRQEIWKA

Contig1275 REYWWALNRKEVWDA

Contig1280 REYWWALNREDVWKA

Contig1281 REYWWELNRQQVWKA

Contig1284 REDWWTANRETVWEA

Contig1285 REYWWALNRNDVWKA

Contig1291 REDWWYANRRQVWKA

Contig1292 REHWWTVNRDQVWQA

Contig1295 REAWWTANRDQVWEA

Contig1299 REDWWNANRDQVWKA

Contig1300 REDWWNANRQQVWKA

Contig1303 REDWWEANRETVWYA

Contig1304 REYWWALNRQTVWKA

Contig1313 REAWWALNRKDVWKA

Contig1318 REDWWEANRQEVWKA

Contig1321 REYWWALNRVQVWNA

Contig1330 REVWWKANRDQVWRA

Contig1332 REDWWALNRNHVWKA

Contig1333 RADWWEANRETVWEA

Contig1334 REDWWYANRAKVWYA

Contig1335 REDWWYANRQEVWKA

Contig1336 REDWWDANRHTVWKA

Contig1337 RNDWWEANRQEIWKA

Contig1339 REAWWDVNRNKVWEA

Contig1343 REYWWEENREKIWKA

Contig1345 REDWWTINRDQVWRA

Contig1348 REDWWNINRKKVWDA

Contig1359 REYWWTANRETVWEA

Contig1362 REDWWYANRATVWKA

Contig1366 REDWWNANRKQIWNA

Contig1367 REDWWNANRLDVWKA

Contig1371 REDWWNANRLDIWKA

Contig1372 REHWWTINREQIWKA

Contig1373 REDWWTLNRRDVWKA

Contig1374 REYWWALNRKDVWKA

Contig1376 REDWWTENRHTVWKA

Contig1380 REDWWEANRETVWKA

Contig1383 REDWWTVNRDQVWKA

Contig1397 REDWWDLNRHDVWKA

Contig1398 REDWWALNRDQVWKA

Contig1400 REDWWELNRQDIWKA

Contig1403 REDWWELNRLEVWKA

Contig1404 REDWWALNRVQVWKA

Contig1406 REYWWALNRRDVWKA

Contig1408 REVWWNVNRDQVWRA

Contig1412 REYWWALNRQEIWKA

Contig1413 REAWWKAHRDQVWKA

Contig1423 REDWWTANRATVWEA

Contig1426 REDWWEANRLQVWKA

Contig1428 REDWWALNRETVWEA

Contig1430 REDWWNANRATVWKA

Contig1432 REAWWTANRNQVWEA

Contig712 REDWWTANRHTVWKA

Contig722 REDWWALNRQEIWKA

Contig768 REDWWTANRHTVWEA

Contig838 REDWWALNRKDVWKA

Contig857 REDWWNANRNDVWKA

Contig863 REYWWEENRETVWKA

Contig899 REDWWDANRATVWEA

Contig939 REDWWNANRNDVWRA

Contig943 REDWWTANRDKVWEA

Contig951 REDWWTANRETVWKA

Contig953 REDWWIANRDQVWEA

Contig962 REDWWTANRDQVWRA

Contig970 REDWWNNNRIMVWYA

Contig972 REYWWALNRQDVWKA

Contig977 REDWWTANRATIWEA

Contig985 REAWWKANRDQVWKA

Contig988 REYWWYANRATIWEA

Contig993 REAWWTINRKDVWKA

Contig995 REAWWNVNRNEVWRA

Contig997 REDWWDANRLDVWKA

Contig998 REDWWALNRQDVWKA
